# 3D-QSAR and Molecular Docking Studies on Derivatives of MK-0457, GSK1070916 and SNS-314 as Inhibitors against Aurora B Kinase

**DOI:** 10.3390/ijms11114326

**Published:** 2010-11-02

**Authors:** Baidong Zhang, Yan Li, Huixiao Zhang, Chunzhi Ai

**Affiliations:** 1 School of Chemical Engineering, Dalian University of Technology, Dalian, Liaoning, 116012, China; E-Mail: zhangbaidong1986@126.com (B.Z.); 2 Center of Bioinformatics, Northwest A&F University, Yangling, Shaanxi, 712100, China; E-Mail: zhx1987619@163.com (H.Z.); 3 Lab of Pharmaceutical Resource Discovery, Dalian Institute of Chemical Physics, Graduate School of the Chinese Academy of Sciences, Dalian, Liaoning, 116023, China; E-Mail: aicy@dicp.ac.cn (C.A.)

**Keywords:** Aurora B, drug design, 3D-QSAR, CoMFA, CoMSIA, molecular docking, homology modeling

## Abstract

Development of anticancer drugs targeting Aurora B, an important member of the serine/threonine kinases family, has been extensively focused on in recent years. In this work, by applying an integrated computational method, including comparative molecular field analysis (CoMFA), comparative molecular similarity indices analysis (CoMSIA), homology modeling and molecular docking, we investigated the structural determinants of Aurora B inhibitors based on three different series of derivatives of 108 molecules. The resultant optimum 3D-QSAR models exhibited (*q*^2^ = 0.605, *r*^2^_pred_ = 0.826), (*q*^2^ = 0.52, *r*^2^_pred_ = 0.798) and (*q*^2^ = 0.582, *r*^2^_pred_ = 0.971) for MK-0457, GSK1070916 and SNS-314 classes, respectively, and the 3D contour maps generated from these models were analyzed individually. The contour map analysis for the MK-0457 model revealed the relative importance of steric and electrostatic effects for Aurora B inhibition, whereas, the electronegative groups with hydrogen bond donating capacity showed a great impact on the inhibitory activity for the derivatives of GSK1070916. Additionally, the predictive model of the SNS-314 class revealed the great importance of hydrophobic favorable contour, since hydrophobic favorable substituents added to this region bind to a deep and narrow hydrophobic pocket composed of residues that are hydrophobic in nature and thus enhanced the inhibitory activity. Moreover, based on the docking study, a further comparison of the binding modes was accomplished to identify a set of critical residues that play a key role in stabilizing the drug-target interactions. Overall, the high level of consistency between the 3D contour maps and the topographical features of binding sites led to our identification of several key structural requirements for more potency inhibitors. Taken together, the results will serve as a basis for future drug development of inhibitors against Aurora B kinase for various tumors.

## 1. Introduction

The Aurora kinases are a family of three highly homologous serine-threonine protein kinases (Aurora A, B and C) that play a critical role in regulating many of the processes that are pivotal to mitosis [1]. Since it was discovered that Aurora kinases are aberrantly over-expressed in various tumor cells [2], there has been intense research in the area of identifying selective Aurora inhibitors as potential drugs; up to now more than 10 small molecules have entered clinical studies [1]. In the last decades, compared with Aurora B, Aurora A has received most of the attention in terms of a link with human cancers in the field of drug development, since the inhibition of Aurora B could rapidly lead to a catastrophic mitosis and cell death, and the inhibition of Aurora B, rather than of Aurora A, is also more crucial for the inhibition of cell proliferation [3].

Aurora B is involved in ensuring chromosome segregation and alignment as part of the chromosomal passenger protein complex (CPC), which plays a key role in regulating progression through and completion of mitosis [4]. A number of studies have characterized the gross cellular effects of disrupting Aurora B in cells, including the expression of kinase dead protein, siRNA depletion of total protein, or microinjection of neutralizing antibodies [1]. Some work also showed that the depression of Aurora B kinase activity by small inhibitors could lead to a failure in cytokinesis and abnormal exit from mitosis, resulting in the endoreduplication, accumulation of polyploidy cells and ultimately apoptosis [5–7].

Encouragingly, series of small molecules have been investigated and exhibited efficient inhibitory activities against Aurora B [4,8–10]. MK-0457, the first Aurora inhibitor to enter clinical trials, can effectively disrupt mitosis and promote apoptosis in cycling cells while still leaving the non-cycling cells unaffected [6]. It also possesses interesting characteristics in that this compound exhibits approximately equal potency to all three types of Aurora kinases, which definitely improves the efficiency of the molecule. GSK1070916 [11], a kind of 7-azaindole derivative, is another potent and selective ATP-competitive inhibitor of both Aurora B and C with a >250-fold selectivity over Aurora A [9]. Recently, this Aurora B inhibitor was also advanced as an agent for the treatment of cancer [12,13]. SNS-314, the third important pan-Aurora inhibitor based on a 4-aminothieno [3,2-d] pyrimidine scaffold, attracted much research interest not only due to its good affinity against all three isoforms of Aurora kinases [1], but also because of its compelling preclinical profile; it has entered clinical trials in patients with solid tumors [4,10].

Structure-activity analysis is the foundation for understanding the structural features of both the inhibitors and the target receptors involved in a particular biological process and thus helps to design more effective inhibitors [14]. Therefore, this method has encouraged its wide use as a rational way to gain insight into the influence of various interactive fields on the activity and thus to aid in the design and forecasting of the inhibitory activity of novel inhibitors [15–21]. In this work, the most widely used computational tools, comparative molecular field analysis (CoMFA) and comparative molecular similarity indices analysis (CoMSIA) methods [22,23], were used to derive 3D-QSAR models for the above three different chemical series of Aurora B inhibitors. Meanwhile, molecular docking was also performed to combine with the 3D-QSAR method, presenting more informative data for the drug design.

To date, a number of Aurora B small molecule inhibitors, from structurally diverse chemical series, have already been reported or reviewed elsewhere [1,4,8–10]. However, very few series of Aurora B inhibitors have so far received much attention from a theoretical perspective. More recently, an elegant 3D-QSAR work concerning the quinazoline derivatives of AZD1152 and ZM447439 classes combined with molecular docking was reported [24]. The authors found the highly active ligands could be designed by varying positively charged, bulky, hydrophobic substitutes at the quinazoline ring, and bulky and hydrophobic groups around the thiazole ring were desirable for higher activity [24]. More recently, several other series of compounds, such as MK-0457 [8], GSK1070916 [9] and SNS-314 [4] derivatives, have been reported as promising Aurora B inhibitors. However, no comprehensive features of the ligand-receptor interactions or detailed structural determinants at the atomic level were obtained for these inhibitors since the X-ray crystallographic structure for the human Aurora B kinase has not been reported to date. Therefore, in the present study, we mainly focus on the study of the above three classes of inhibitors with an attempt to disclose the structural features of anticancer Aurora B inhibitors using an integrated computational method including 3D-QSAR, homology modeling and molecular docking simulations. A comparison was also performed to identify similarities and differences in the binding modes for each class, and thus a set of vital amino acid residues were found to play a critical role in stabilizing the ligand-receptor interactions of Aurora B kinase. To our knowledge, this work presents the first 3D-QSAR study for these series of compounds, which will provide a platform for the screening and design of novel Aurora B inhibitors as important weapons in the fight against tumors.

## 2. Materials and Methods

### 2.1. Data Sets

All molecules used as Aurora B inhibitors in the present study have been collected from the literature recently published [4,8–10]. Discarding compounds with unspecified inhibitory activity, the data set used comprises series of diverse MK-0457, GSK1070916, SNS-314 derivatives, which have been shown to possess a wide spectrum of inhibitory activities against Aurora B enzyme. The three different groups of compounds were assayed for their Aurora B inhibitory activity by using the standard coupled enzyme assay [8], the human lung cancer cell line A549 [9], the humanized mouse Aurora enzyme [10], Aurora B enzymatic assay and a BrdU cell proliferation assay [4]. The *in vitro* biological activities *K*_i_ (μM) and the *IC*_50_ values (μM) were converted into the corresponding inhibitory activity p*K*_i_ (−log*K*_i_) and p*IC*_50_ (−log*IC*_50_) values as dependent variables in deriving the QSAR models. Since the 3D-QSAR models were generated from training set molecules and further confirmed using an external test set, each group was divided into two sets, consisting of training and tested compounds. The test set was selected in such a way that the experimental values are almost uniformly distributed in the range of the values for the whole set. The structures and inhibitory activity data of representative compounds in the training and test sets are given in Tables 1–3. (All the chemicals with their structures, biological values, and their division into the training and test sets are listed in the supporting information).

### 2.2. Molecular Modeling

All the 3D-QSAR and molecular docking computations were performed using Sybyl (Tripos, Inc.) [25]. The 3D structures of molecules were built using the Sketch Molecule function with Sybyl software. The geometry optimizations of all compounds were carried out by using the TRIPOS force field with the Gasteiger Huckel charges, and repeated minimization was performed using Powell conjugated gradient algorithm method until the root-mean-square (rms) deviation of 0.001 kcal/mol was achieved. In the present study, the most potent molecule of each class (compounds 25, 40, 105, respectively) was chosen as a template to fit the remaining compounds in the training and test sets through the fit atoms function in SYBYL. Thus, all compounds finally minimized with the lowest energy in the data set were aligned to a common substructure by substructure-based alignment method using the “align database” command in SYBYL. The determined common substructures for the alignment are shown in bold face. (See Tables 1–3).

### 2.3. 3D-QSAR Analysis

To derive the CoMFA and CoMSIA descriptor fields, a 3D cubic lattice with grid spacing of 2 Å in *x*, *y*, and *z* directions, was finally generated to encompass the aligned molecules. In CoMFA, descriptors of steric and electrostatic fields were calculated using an sp^3^ carbon probe atom with a van der Waals radius of 1.52 Å and a charge of 1.0 to generate energies for both the steric and electrostatic fields with a distance-dependent dielectric at each lattice point. Energy values for both steric and electrostatic fields were truncated at a default energy cut-off value of 30 kcal/mol. The CoMFA steric and electrostatic fields generated were automatically scaled using the CoMFA-STD method in SYBYL. Another 3D QSAR procedure, CoMSIA, involving a common probe atom and similarity indices calculated at regularly spaced grid intervals for the prealigned molecules, were derived with the same lattice box implemented in SYBYL as that used for the CoMFA calculations. In addition to steric and electrostatic fields, hydrophobic, and hydrogen-bond donor and acceptor descriptors were calculated with the same lattice box of a regularly placed grid of 2.0 Å, employing a probe atom with radius 1.0 Å, charge 1.0, and hydrophobicity +1.0. CoMSIA similarity indices (*A*_F_) for a molecule *j* with atoms *i* at a grid point *q* were calculated by Equation (1):

(1) $$A_{\text{F},\text{K}}^{q}(j)=-\sum \omega_{\text{probe},k}\omega_{ik}e_{iq^{2}}^{-\text{ar}}$$

where *k* represents the steric, electrostatic, hydrophobic, hydrogen-bond donor or hydrogen-bond-acceptor descriptor. Compared to the CoMFA approach, which has two fields, in the CoMSIA method, five physico-chemical properties were associated, including three additional properties of hydrophobic, hydrogen bond donor and hydrogen bond acceptor, which were evaluated using the common sp^3^ carbon probe atom. Meanwhile, a default value of 0.3 was used as the attenuation factor and a distance dependent Guassian type functional form has been used between the grid point *q* and each atom *i* in the molecule. This can avoid singularities at the atomic positions and the dramatic changes of potential energy due to grids in the proximity of the surface [26].

In the partial-least-squares (PLS) analysis, the CoMFA and CoMSIA descriptors served as independent variables and the p*IC*_50_ or p*K*_i_ (μM) values served as dependent variables to deduce 3D-QSAR models [27–31]. The predictive capabilities of the models were first evaluated in leave-one-out (LOO) cross validation method. The number of components resulting in the highest cross-validated *r*^2^ and lowest standard error of prediction (*SEP*) was determined as the optimum number of principal components (Nc) in the final PLS analyses. The predictive *r*_pred_^2^ based on molecules in the test set was calculated to evaluate the predictive power of the CoMFA and CoMSIA models using Equation (2):

(2) $$r_{\text{pred}}^{2}=(SD-PRESS)/SD$$

where *SD* is the sum of the squared deviations between the actual activities of the molecules in the test set and the mean activity of the molecules in the training set, and *PRESS* is the sum of the squared deviations between the predicted and the actual activity values of every molecule in the test set.

### 2.4. Homology Modeling

Homology modeling procedures are indispensable tools for conducting research involving structure based drug design when the experimental 3D-structure of the receptor is not available [32]. In the present study, due to the unavailability of Aurora B X-ray crystallographic structure for humans, homology modeling process was employed as a theoretical method to predict the protein structure from the target amino acid sequence (accession BC000442) obtained from the National Center for Biotechnology Information database (http://www.ncbi.nlm.nih.gov). The homology model of Aurora B was built based on sequence alignment and the obtained target amino acid sequence was submitted to SWISS-MODEL server (Automated Comparative Protein Modeling Server, Version 3.5, GlaxoWellcome Experiment Research, Geneva, Switzerland, http://swissmodel.expasy.org) [33,34] for a comparative structural modeling. Meanwhile, the template protein (PDB code 2BFX chain A from Protein Data Bank http://www.rcsb.org), which exhibits a high resolution (1.8 Å), was employed to generate the 3D protein structure. All hydrogen atoms were subsequently added to the unoccupied valence of heavy atoms at the corresponding neutral state using the biopolymer module of SYBYL package.

### 2.5. Molecular Docking

To explore the interaction and illustrate the accurate binding model for the active site of Aurora B with its ligands, molecular docking analysis was carried out by using the Surflex Dock implemented in SYBYL. Meanwhile, the resulting homology protein structure for docking was further developed using the protein preparation and refinement utility provided by SYBYL. Finally, each conformer of all 108 inhibitors in three different groups was docked into the binding site 10 times. Prior to docking analysis, in order to assure the quality of the binding mode of the ligands and reproduce the proper X-ray structure, the following criteria were applied to perform molecular docking analysis: (1) The key residues like Glu161 and Ala157, as major contributors to the enhanced affinity [35], should well bind to ligand; (2) the most potent inhibitors (compounds 25, 40 and 105) should have similar binding poses in the active site and the top ranked docked solution in one favorable cluster of docking poses meets satisfying root-mean-square deviation (RMSD) values; (3) the putative poses of the potent compounds were also scored using the Hammerhead scoring function [36], which also serves as an objective function for local optimization of poses. Additionally, two parameters, *i.e.*, protomol_bloat and protomol_threshold, which determine how far from a potential ligand the site should extend and how deep into the protein the atomic probes used to define the protomol can penetrate, are specified 1_0.55, 0_0.66 and 0_0.75 for each group, respectively.

## 3. Results and Discussion

### 3.1. CoMFA and CoMSIA Statistical Results

In order to develop an effective model with good prediction, a number of parameters, such as the cross-validated correlation coefficient (*r*^2^_cv_), non-cross-validated correlation coefficient (*r*^2^_ncv_), standard error estimate (*SEE*) and *F*-statistic values were taken into consideration. For all of the 3D-QSAR models, the LOO cross-validation was performed first to identify the cross-validated correlation coefficient (*q*^2^) values. Then the number of components identified in the LOO cross-validation process was used in the final non-cross-validated PLS run. Generally, a *q*^2^ value of greater than 0.5 is usually considered significant. To further assess the stability and confidence of the derived CoMFA and CoMSIA models, bootstrapping analysis for 100 runs was applied to the compounds of the training set. In CoMSIA, five descriptors (steric, electrostatic, hydrophobic, and hydrogen-bond-donor and hydrogen-bond-acceptor) are available to be considered. But it has been established that the five different descriptor fields are not totally independent of each other and that such dependency among individual field usually decrease the statistical significance of the models [37]. For this reason, all 31 possible descriptors’ combinations for each group were calculated with purpose to build the optimal 3D-QSAR models with the highest *q*^2^ values and other statistical results for each class. Table 4 summarizes the statistical results of the optimum model for each class, and for the modeling results of the other 93 combinations of CoMFA or CoMSIA descriptors, see Tables S7–S9 in supporting information.

### 3.2. Validation of the 3D QSAR Models

**Figure 1.**
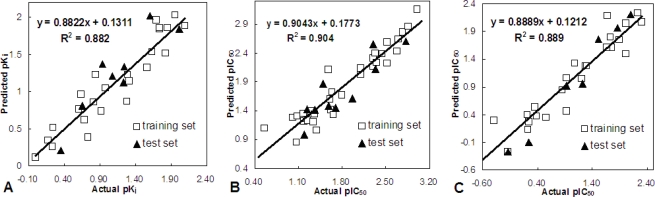
The correlation plots of predicted *versus* actual Aurora B inhibitory activities using the training (white squares) and test (black triangles) sets based on (**A**) CoMSIA model of MK-0457; (**B**) CoMSIA model of GSK1070916 and (**C**) CoMSIA model of SNS-314. The solid lines are the regression lines for the fitted and predicted bioactivities of training and test compounds in each class, respectively.

Statistically significant CoMFA and CoMSIA models were derived from the training compounds and further used to predict test molecules. The resultant optimum models exhibited agreeable statistical results of (*q*^2^ = 0.605, *r*^2^_pred_ = 0.826), (*q*^2^ = 0.52, *r*^2^_pred_ = 0.798) and (*q*^2^ = 0.582, *r*^2^_pred_ = 0.971) for MK-0457, GSK1070916 and SNS-314 classes, respectively (Table 4), and relatively small prediction errors (<−0.098, 0.044 and 0.038, see Supporting Information). The experimental *versus* predicted activities are shown in Figure 1, through which we can find that all the training and test compounds are well distributed around the regression lines, indicating that the obtained CoMFA/CoMSIA models presented good performance on both the training and test compounds.

#### 3.2.1. MK-0457

The 3D-QSAR models were generated from MK-0457 derivatives with p*K*_i_ (μM) values ranging from 0.002 to 2.097 (24 training and 8 test compounds). The statistical results of the optimal model are in Table 4. Satisfyingly, most of the 31 models derived from various combinations of fields present high predictive *r*^2^_cv_ values (>0.5). (See Supporting Information). The optimal CoMSIA model yielded *r*^2^_cv_ = 0.605 with 3 components, *r*^2^_ncv_ = 0.882, *r*^2^_pred_ = 0.826 and the respective steric and electrostatic field contributions of 33% and 67%. And the best CoMFA model also presented reasonable statistical features with *r*^2^_cv_ = 0.604, 8 components, *r*^2^_ncv_ = 0.992, *r*^2^_pred_ = 0.692, steric 62% and electrostatic 38% field contributions. Overall, the performance of the CoMSIA model is superior to that of the CoMFA one. Meanwhile, an incorporation of the hydrogen-bond donor/acceptor or both fields yielded makes the models perform poorer, suggesting the steric and electrostatic fields were statistically robust in building the models.

#### 3.2.2. GSK1070916

Table 4 summarizes the statistical parameters of the optimal model for GSK1070916 compunds. A combination of steric and electrostatic fields produced poor CoMFA and CoMSIA models with internal predictions of *r*^2^_cv_ = 0.295 and 0.178, respectively. While, incorporation of the hydrogen-bond donor/acceptor or both fields could improve the model performance, thus the optimal CoMSIA model generated with these fields showed a reasonable *r*^2^_cv_ = 0.52 with 4 components, *r*^2^_ncv_ = 0.904, *r*^2^_pred_ = 0.798 and higher electrostatic field contribution (69%) than hydrogen-bond-donor (31%) field. Meanwhile, the models derived from the combinations of SDA (steric, hydrogen-bond donor and acceptor fields) and HDA (hydrophobic, hydrogen-bond donor and acceptor fields) showed comparable predictions. However, both of them were not accepted as they applied more number of the components (up to 9). The 3D contours analyzed for the generated model are shown in Figure 4 (A, B).

In building the models, compound 35 was treated as an outlier, because including this compound the optimal models yielded a high residual value of more than 1 logarithm unit. In addition, the PLS analysis on alignment of all the compounds resulted in modest *r*^2^_cv_ values (averagely < 0.30), indicating possible outlier exists in this data set. This outlier might be due to experimental errors since this compound has a similar functional group in –R_3_ group with those less active compounds, such as 48, 52, 53 and 54, while this compound has a high p*IC*_50_ value.

#### 3.2.3. SNS-314

Selective SNS-314 Aurora B inhibitors with *IC*_50_ (μM) values ranging from 0.005 to 5.600 were used to generate 3D-QSAR models (24 training and 7 test compounds). Although these compounds were retrieved from two independent publications [4,10], three common compounds (77, 80 and 83) were found to be in both literatures with exactly the same biological activities, which further validates the feasibility of utilizing the multi-source data. The statistical parameters of the optimal model are shown in Table 4. The CoMFA model showed poor internal predictions (*r*^2^_cv_ = 0.079) using steric and electrostatic fields, which is also true for the CoMSIA model. However, the models using a combination of SEHD could improve the model performance (*r*^2^_cv_ = 0.069–0.430, *r*^2^_ncv_ = 0.555–0.716 and *r*^2^_pred_ = 0.806–0.937). And the model obtained with combination of hydrophobic and hydrogen-bond donor fields showed highest *r*^2^_cv_ = 0.582, *r*^2^_pred_ = 0.971, *r*^2^_ncv_ = 0.910 and the corresponding contributions of hydrophobic and hydrogen-bond-donor fields of 60.7% and 39.3%. Therefore, this model was further used to analyze 3D contour plots in Figure 5 (A, B).

Compound 107 was eliminated in building the models, as the best model with this compound produced a modest *r*^2^_cv_ value of 0.385. Omission of this resulted in a great increase in *r*^2^_cv_ value to 0.582. The outlier status of compound 107 could stem from its structural uniqueness, when compared to its counterparts, compounds 94–106.

### 3.3. Homology Modeling

**Figure 2.**
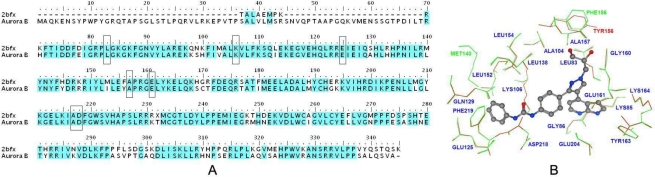
(**A**) The alignments of the sequences of 2BFX chain A template and Aurora B target protein. The identical amino acid residues in the sequence alignment are highlighted in cyan. Dashed lines denote the amino acid residues deletion. The key residues of binding site are highlighted in black rectangles; (**B**) The enlargement of the superposition structure of the active site with compound 40 displayed in sticks. The residues from the template protein and the homology modeling protein are highlighted in green and red colors respectively, the same residues in the active site are labeled in blue color, while the residues differing between them are labeled in their own color.

The initial sequence alignment between the target (Aurora B kinase for humans) and the template (PDB code: 2BFX) sequences is shown in Figure 2A. The whole sequence identity between the target and the template protein is 80.6% and therefore, we conclude that this alignment can be used to construct a reliable 3D model [38]. Additionally, besides the insertions and deletions detected in the loop regions corresponding to the functional regions of Gly loop (amino acids 81–93), catalytic cleft (amino acids 154–161) and activation loop (amino acids 80–220) [35], there is only one single replacement detected in the catalytic cleft and two in the activation loop region, and thus the identity of the functional region is as high as 85.0%. The superposition of the two 3D structures is shown in Figure S1, indicating that the overall conformation of the modeling target is very similar to the template with a root-mean-square deviation (RMSD) of 0.078 Å. In addition, our alignment was also carefully checked in the key residues of binding site (highlighted in black rectangles) where it was found that all critical amino acids (such as Leu83, Lys106, Glu125, Ala157, Glu161 and Asp218) were well overlaid in 3D space in the two structures (Figure 2B).

### 3.4. Validation of the Docking Protocol

**Figure 3.**
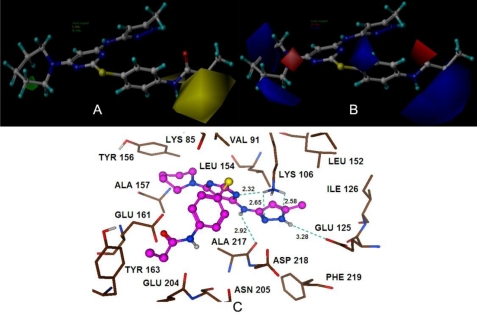
CoMSIA StDev*Coeff contour plots for MK-0457. (**A**) The steric (green/yellow) contour map represents respective 95% and 5% level contribution combined with compound 25. Green contours indicate regions where bulky substituents increase activity; yellow contours indicate regions where bulky substituents decrease activity; (**B**) The electrostatic (red/blue) contour map represents respective 75% and 25% level contribution combined with compound 25. Red contours indicate regions where negative charged substituents increase activity; blue contours indicate regions where positive charged substituents increase activity; (**C**) The enlargement for stereoview of the docking structure of compound 25 in complex to the active site of the monomer structure of the Aurora B. Hydrogen bonds are shown as dotted green lines. Active site amino acid residues are represented as sticks, while the inhibitor is shown as ball and stick model.

**Figure 4.**
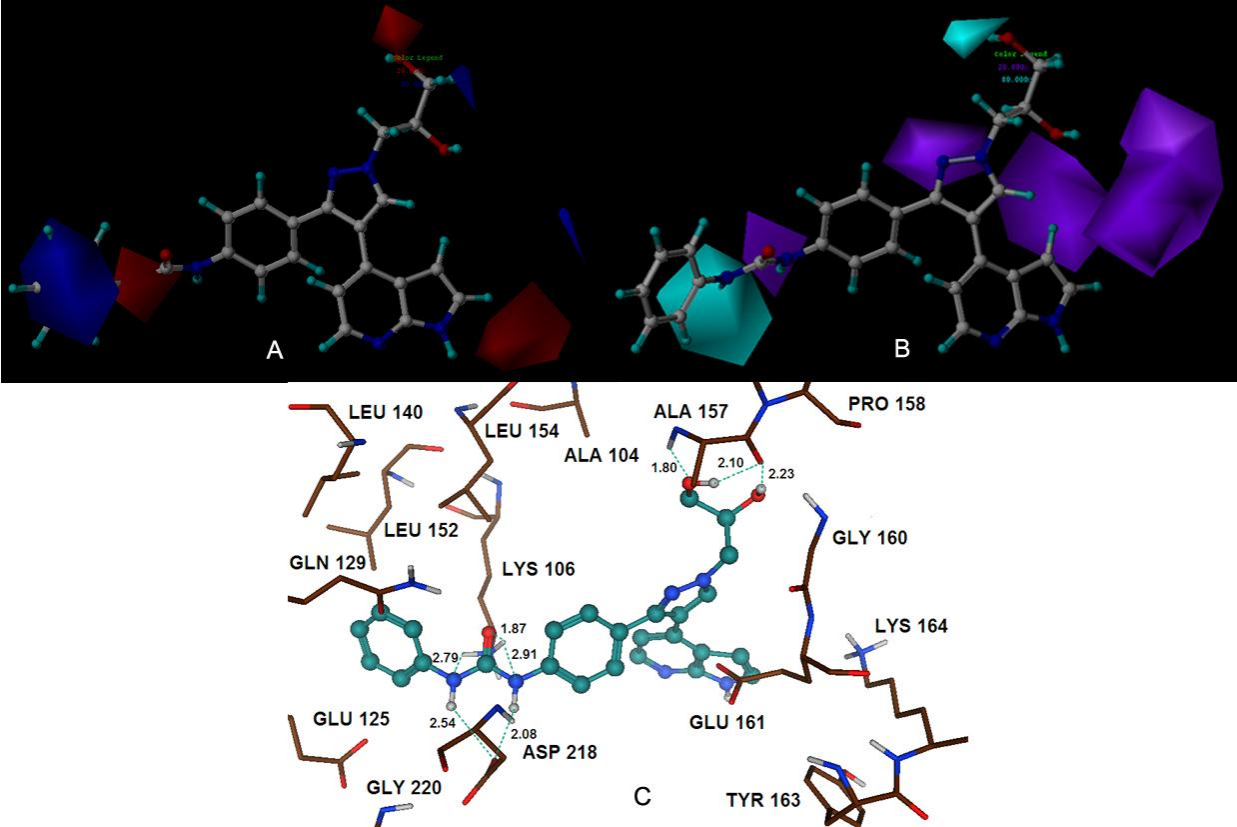
CoMSIA StDev*Coeff contour plots for GSK1070916. (**A**) The electrostatic contour map (red/blue) represents respective 80% and 20% level contribution combined with compound 40. Red contours indicate regions where negative charged substituents increase activity; blue contours indicate regions where positive charged substituents increase activity; (**B**) The hydrogen-bond donor (cyan/purple) contour map represents respective 80% and 20% level contribution combined with compound 40. Cyan contours indicate regions where hydrogen-bond-donor favorable substituents increase activity; purple contours indicate regions where hydrogen-bond-donor unfavorable substituents decrease activity; (**C**) The enlargement for stereoview of the docking structure of compound 40 in complex to the active site of the monomer structure of the Aurora B. Hydrogen bonds are shown as dotted green lines. Active site amino acid residues are represented as sticks, while the inhibitor is shown as ball and stick model.

**Figure 5.**
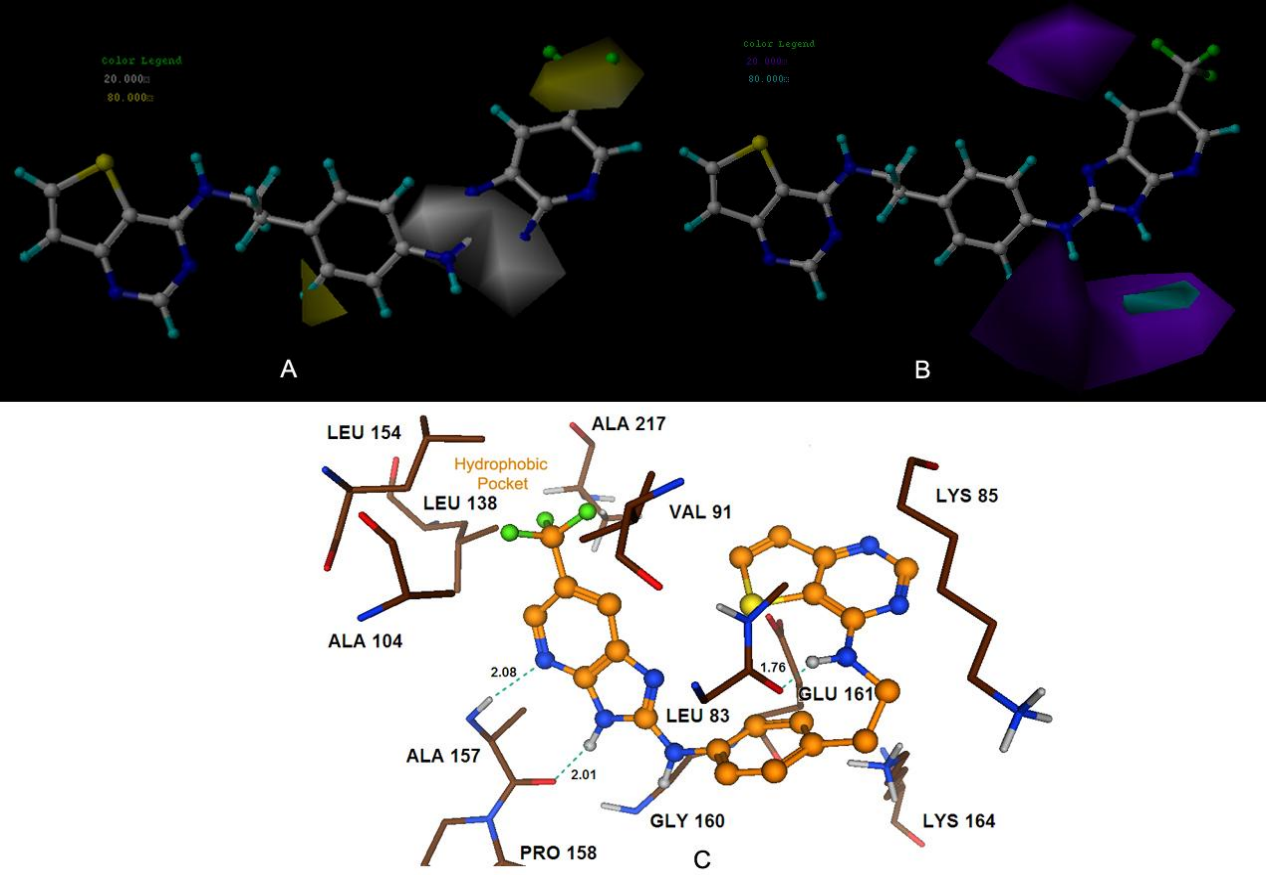
CoMSIA StDev*Coeff contour plots for SNS-314. (**A**) The hydrophobic contour map (yellow/white) represents respective 80% and 20% level contribution combined with compound 105. Yellow contours indicate regions where hydrophobic favorable substituents increase activity; white contours indicate regions where hydrophobic unfavorable substituents increase activity; (**B**) The hydrogen-bond donor (cyan/purple) contour map represents respective 85% and 15% level contribution combined with compound 105. Cyan contours indicate regions where hydrogen-bond-donor favorable substituents increase activity; purple contours indicate regions where hydrogen-bond-donor unfavorable substituents decrease activity; (**C**) The enlargement for stereoview of the docking structure of compound 105 in complex to the active site of the monomer structure of the Aurora B. Hydrogen bonds are shown as dotted green lines. Active site amino acid residues are represented as sticks, while the inhibitor is shown as ball and stick model.

Docking calculations were used to find the optimal conformation of the ligand in the binding pocket of Aurora B protein. The top ranked docked solution of each group was found in one favorable cluster of docking poses with an average RMSD value 0.61 Å, 0.03 Å and 1.37 Å, respectively, demonstrating the binding mode is correctly reproduced. Additionally, the putative poses of the potent compounds were scored using the Hammerhead scoring function, which serves as an objective function for local optimization of poses. During this docking process, the protein was considered to be rigid, while the ligands flexible. By this process, we found that the binding modes for the most potent compounds of each class presented statistically significant total score results of 5.89, 6.19 and 4.98, respectively. The most active inhibitors of each group have been nicely docked to the active site and the docked models (compounds 25, 40 and 105) are shown in Figures 3C, 4C and 5C, respectively.

### 3.5. 3D-QSAR Contour Maps and Molecular Docking Correlation

#### 3.5.1. MK-0457

The steric and electrostatic fields of CoMSIA are depicted in Figure 3 (A, B). Compound 25, the most potent inhibitor in this series, was overlaid as a reference structure on the maps. The steric contour map showed a green region at –R_2_ group and this substituent partially extended outside the binding pocket (shown in Figure 3C), indicating the requirement of bulky substituents in this region for a potent Auora B inhibitor. This may account for the qualitative SAR observation that compounds 24–31, with the introduction of heterocycles as the 6-substituent on the pyrimidine, had an inhibitory improvement against Aurora B [8]. Therefore the low potency of compounds (18, 19 and 20) can be explained as they have much smaller groups, such as methyl, cyclo-propyl, tert-butyl, respectively. Meanwhile, a sterically disfavored yellow contour is present at the –R_3_ group, which strongly delimits the sideward relocatability. Interestingly, the docking study lends further support to the concept that this area was occupied by the residues of Glu161 and Tyr163, indicating that bulky substituents at this position will conflict with these residues and decrease the activity (shown in Figure 3C). This is reflected in compounds 8, 9 and 11, which have bulky substituents (–NHSO_2_Me, –NHC(O)OtBu, –NMeC(O)Me), respectively, at this position with pK_i_ values below 1. This can serve as an explanation for the higher activities of compounds 24–31, who have more bulky substituents in the green regions and less bulky substituents in the yellow regions.

For the electrostatic contour maps shown in Figure 3B, positive charges favored regions depicted by blue are found on both sides of the –R_2_ group, suggesting that positive charged groups are appreciated here. Therefore, it can be explained that the presence of the residues Ala157 and Glu161 observed appearing adjacent to these regions. Another blue contour observed beside the –R_2_ group may possibly account for the low activity of compounds 8, 9, which have substituents of –SO_2_– and –C(O)O–, respectively, right in the blue region. In addition, a red contour at atom N of the piperidine ring suggests that a negative charged substituent at this position will enhance inhibitory potency. A comparison of compounds 25 and 21–23 shows that a change from a carbon atom to a nitrogen atom of the aromatic ring greatly increases the potency, which may be due to a negative charge increase at this position.

#### 3.5.2. GSK1070916

The graphical representation for the CoMSIA model from electrostatic and hydrogen-bond donor fields is depicted in Figure 4 (A, B). Compound 40 was applied as a reference. The blue contour completely enclosed the phenyl ring which specifies that positively charged substitutents in this region may increase the activity. This is consistent with the docking study that the phenyl ring is surrounded by the amino acids Gln129, Glu125 and Asp218.

The medium size red-colored contour located reside the pyrrole ring indicates the significance of less positive charged substituents in this region. Considering compounds 55, 57, 62, 63, both the two molecules of 55, 62 as well as 57, 63 have the same structures except at the 1-position of –R_2_ group. The reason for the difference in activities is attributed to the extra carbonyl substituents of compounds 55 and 57 at this position. Therefore, enhanced activity might be obtained if a negatively charged group is added to this position. The CoMSIA contour map for electrostatic field also has a red contour enclosing the –NH– at the 2-position of –R_3_ group, which indicates that the negative charged substituents are preferred for higher activity. Meanwhile, a cyan contour of hydrogen-bond-donor field located at the same position suggests the structural requirement for hydrogen-bond-donor favorable substituents. These findings point to the need for electronegative groups with hydrogen bond donating capacity, such as –NH–, which will probably increase the biological activity. Furthermore, this is also consistently reflected in the docking study shown in Figure 4C; the –NH– playing a key role as a hydrogen bond donor was involved in hydrogen bonding interactions with the backbone of Asp218 (2.73 Å, 122.6°and 2.54 Å, 135°). Similarly, both the red and cyan small contours were observed appearing adjacent to the hydroxyl of –R_1_ group, thus suggesting that a negatively charged substituent with hydrogen bond donating capacity added to this position would engage in interactions with the receptor and enhance the inhibitory activity. As expected, Ala157 amino residue was found to form strong hydrogen bond contacts with the hydroxyl of compound 40 (1.80 Å, 142.5° and 2.10 Å, 157.2°). Consequently, hydroxyl of –R_1_ group appears to plays an important role in stabilizing the ligand-receptor interactions. Moreover, these findings further support the putative binding mode of the initial structure-activity relationship study that the pyrazole ring occupies the sugar pocket region of the ATP-binding site [14]. Therefore, this may possibly account for the high Aurora B inhibitory activity of compounds 39, 40 and 41, which have incorporated polar hydrogen bond donating groups (–OH) forming hydrogen bonds with Ala157 residue to enhance the potency.

#### 3.5.3. SNS-314

Figure 5 provides the graphical representation for the CoMSIA model using hydrophobic and hydrogen-bond donor fields, with compound 105 as the template. The yellow contour at the –R_3_ group suggests that substituents added here desired a favorable hydrophobic interactions with the target receptor. This is consistent with the docking study that most of the amino acid residues near the yellow contour regions are hydrophobic in nature (e.g. Val, Ala and Leu). As depicted in Figure 5C, the substituent (–CF_3_) at the –R_3_ group is placed in the hydrophobic pocket formed by Leu154, Leu138, Val91, Ala104, and Ala217. Thus, this can be expected to explain the correspondingly lower activities of compounds 80 and 89. In contrast, compounds 82 and 88 that have groups with high hydrophobicity, such as –CF_3_ and –F, at the –R_3_ group of the aromatic ring are distinctly more active [4]. Another small yellow contour observed close to the meta-position of the phenyl ring indicates that hydrophobically favored substituents connected to this position will enhance the biological activity. For example, the structure of compound 100 has an N atom at meta-position of the phenyl ring, while compound 103 has a C atom in the opposite and thus shows a distinctly less inhibitory activity than compound 100. Meanwhile, the white contour observed encompassing the imidazole ring moiety indicates the significance of hydrophilic substituents here. This is in agreement with the experimental observation that compounds 97–99 with more hydrophobic substituents of –NMe–, –O–, –S–, respectively, in this region have a lower activity than compound 94. The reasonably higher inhibitory activity of compound 94 is probably due to occupancy of –NH–, which is placed in the white contour and forms hydrogen bond with residue identified as Ala157 (2.03 Å, 162.5°). A small cyan contour seen distantly located from the –NH of the imidazole ring suggests occupancy of this spatial region by a hydrogen bond donor group for a strong inhibitory activity. This may be due to the involvement of Ala157 which plays a major role as a hydrogen bond acceptor during the interaction with target. A medium size purple contour map seen under the phenyl ring indicates that the region is preferred to hydrogen bond acceptor groups. And this observation is also consistent with our previous docking study that indicated that –NH of the Gly160 residue acting as a hydrogen-bond donor at this area would create desirable close contact between the receptor and the ligand as shown in Figure 5C.

### 3.6. Comparison of Binding Modes for Each Class

In order to get a better understanding of the variations in biological activities, we compared the binding modes of each group seeking to explore their similarities and differences. Our docked models revealed that hydrogen bonding is an important interaction between the inhibitor and the target receptor. According to the docking study, a total of five hydrogen bonds were formed between compound 25 and residues Lys106 (2.32 Å, 153.8°; 2.65 Å, 117.2°; 2.58 Å, 114.8°), Ala217 (2.92 Å, 101.5°), and Glu125 (3.28 Å, 160.2°) of the target receptor (Figure 3C). Interestingly, the common structure of MK-0457 derivatives was found to form a total of five key hydrogen bonding interactions with the receptor. Therefore, this further supports the evidence of its essential role for the overall inhibitory activity. Furthermore, Ala157 and Glu161 residues were also found to possess important electrostatic repulsion interactions with the ligand. Additionally, for the derivatives of GSK1070916, eight hydrogen bonds were uncovered during the docking procedure. Amino acids of Lys106 (1.87 Å, 129.6°; 2.79 Å, 110°; 2.91 Å, 119.8°), and Asp218 (2.08 Å, 150.3°; 2.54 Å, 135°) appeared to have hydrogen bonding interactions with compound 40. Meanwhile, residue Ala157, identified as a major contributor to the enhanced affinity, was also uncovered to form strong hydrogen bonds with the receptor (2.10 Å, 157.2°; 1.80 Å, 142.5°; 2.23 Å, 166.6°). For this reason, it provided stable interactions of inhibitors with the surrounding environment. In addition, for the class of SNS-314, only three hydrogen bonds were formed between the active binding site of the target receptor and the docked compound 105. As depicted in Figure 5C, Ala157 (2.01 Å, 163.3°; 2.08 Å, 161.7°) and Leu83 (1.76 Å, 146.3°) were involved in the hydrogen bonding contacts with compound 105, possessing a further stabilization between the ligand and the receptor.

**Figure 6.**
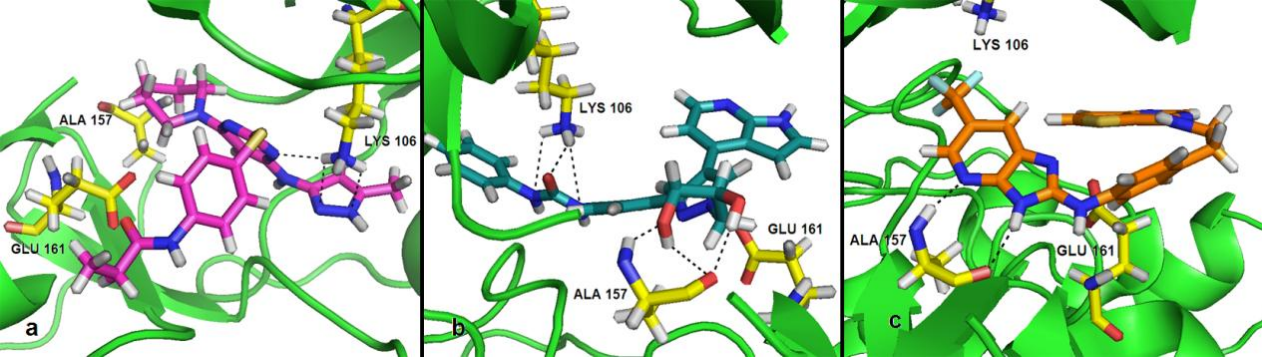
Stereoview of the docked conformations of compounds 25, 40 and 105, respectively, in the active site of Aurora B kinase. The hydrogen bonds are shown by broken lines. Compounds 25, 40 and 105, colored purple, cyan and orange, are presented in pictures a, b and c, respectively. The important amino acid residues, Lys106, Ala157 and Glu161 (stick rendering) are colored by atom type (C, yellow; N, blue; H, white; O, red).

Interestingly, two common active amino acid residues were found among the three classes (as shown in Figure 6). Lys106 residue was found to possess hydrogen bonding interactions with both the inhibitors (compounds 25 and 40), respectively, whereas, compounds 40 and 105 both presented Ala157 as an active amino acid residue. Therefore, it can be reasonably presumed that Ala157 and Lys106 are considered to be vital amino acids that have great effects on the ligand-receptor interactions of Aurora B kinase. Therefore, it may possibly account for the overall higher inhibitory activities of GSK1070916 class than the MK-0457 and SNS-314 classes. The most potent inhibitor of GSK1070916 derivatives (compound 40) has more hydrogen bonding interactions with both Ala157 and Lys106 residues and thus is more active than the other two inhibitors (compounds 25 and 105) that do not. Additionally, the docking study also revealed the importance of the amino acid esidue, Glu161, which possesses strong electrostatic repulsion interactions with all the three potency inhibitors.

## 4. Conclusions

The 3D-QSAR studies yielded stable and statistically significant predictive models with relative high cross-correlation coefficients for predicting the activities of new Aurora B inhibitors. A high LOOCV *r*^2^ value and a small standard deviation indicate the existence of a similar relationship in all compounds of the series used in the study. The overall study for the optimal model from the MK-0457 class implies the crucial roles of steric and electrostatic field effects, while the GSK1070916 model revealed the importance of electrostatic and hydrogen-bond donor fields. In addition, for SNS-314, hydrophobic and hydrogen-bond donor fields were found to be more important than the other descriptors.

Satisfyingly, a good correlation was attained between the 3D-QSAR contour maps and the corresponding predictive binding mode. For the MK-0457 model, the bulky substituent of –R_2_ group plays a main contribution toward the inhibitory activity, which is consistent with the existence of a wide steric gorge enclosing this group. In addition, the carbonyl group at 1-position is critical for the increase in the inhibitory activity. For GSK1070916 compounds, the preference for electronegative groups with hydrogen bond donating capacity at 2-position and –R_1_ group shows a great impact on the overall inhibitory activities. The model for SNS-314 revealed the hydrophobic favorable property at the –R_3_ group, which is consistent with the docking results. And the docking analysis demonstrated the importance of Glu161, Ala157 and Lys106 in facilitating Aurora B recognition of its inhibitors.

## Supplementary Data



## Figures and Tables

**Table 1.** Representative skeletons and molecular structures of MK-0457 derivatives and their binding affinity values (p*K*_i_). 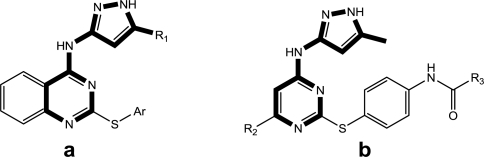

**Compound**	**Template**	**R_1_**	**Ar**	**p*K*_i_(μM)**
8	a	Me	4-(NHSO_2_Me)Ph	0.638
9	a	Me	4-(NHC(O)OtBu)Ph	0.602
11	a	Me	4-(NMeC(O)Me)Ph	0.979

**Compound**	**Template**	**R_2_**	**R_3_**	**p*K*_i_ (μM)**
18	b	Me	Et	0.815
19^a^	b	CyPr	Et	1.229
20^a^	b	tBu	Et	0.939
21	b	Ph	Et	0.839
22	b	3-Py	Et	1.284
23	b	4-Py	Et	1.310
24	b	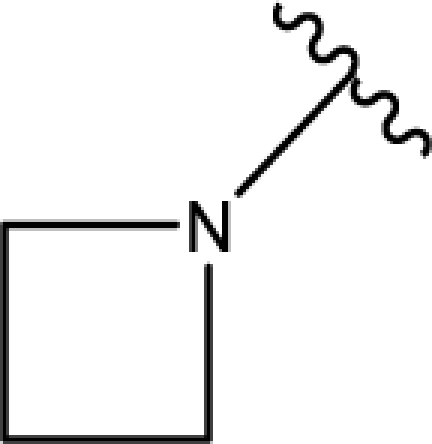	Et	1.638
25	b	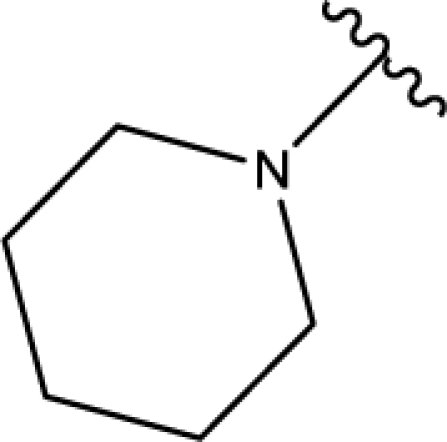	Et	2.097
26	b	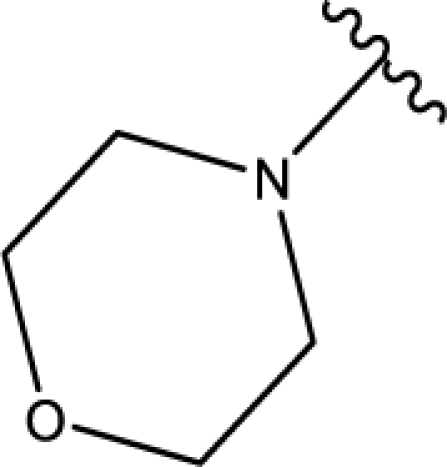	Et	1.854
27	b	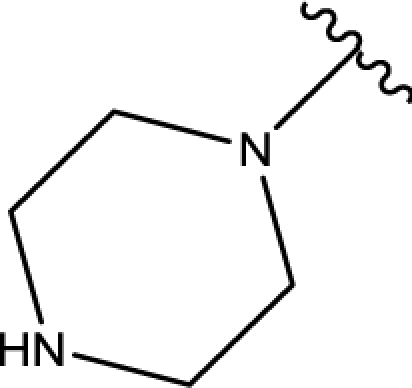	Et	1.745
28	b	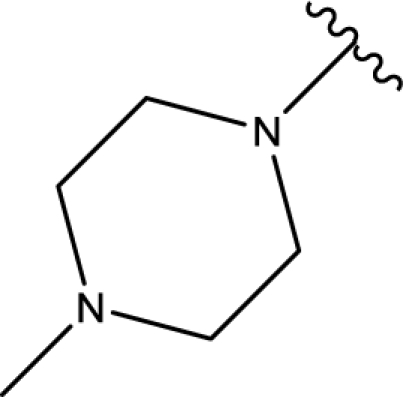	Et	1.699
29^a^	b	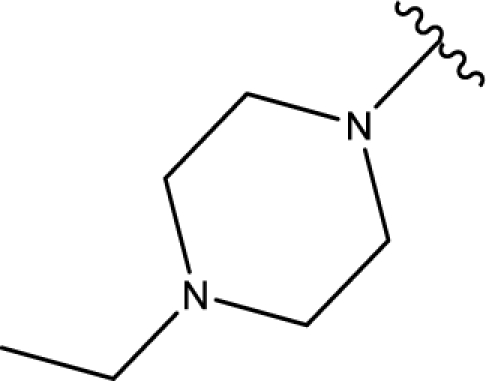	Et	1.602
30^a^	b	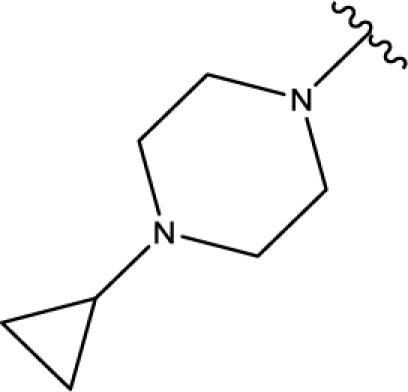	Et	2.022
31	b	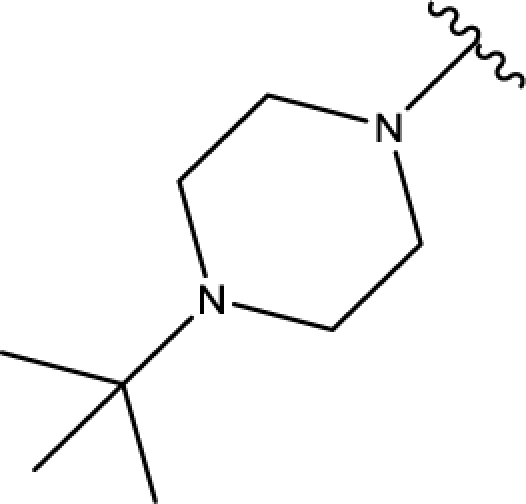	Et	1.959

a Test set molecules. The common structure for molecular alignment is shown in bold face.

**Table 2.** Representative skeletons and molecular structures of GSK1070916 derivatives and their binding affinity values (p*IC*_50_). 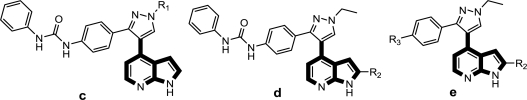

**Compound**	**Template**	**R_1_**		**p*IC*_50_ (μM)**
35^b^	c	Et		2.699
40	c	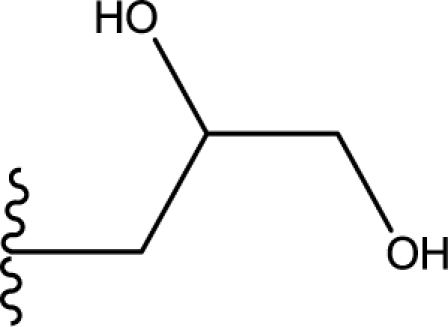		3.000
41	c	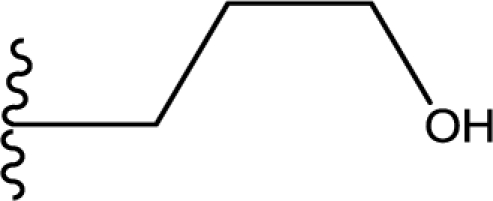		2.699

**Compound**	**Template**	**R_2_**		**p*IC*_50_ (μM)**
48	d	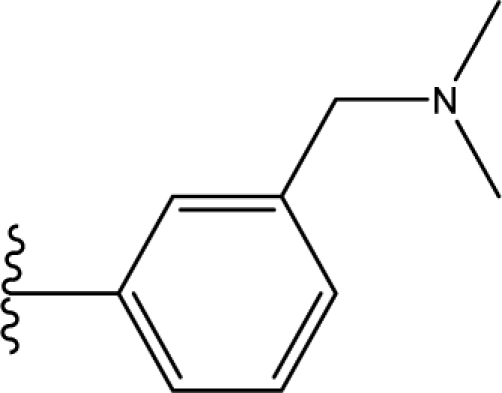		1.699
52	d	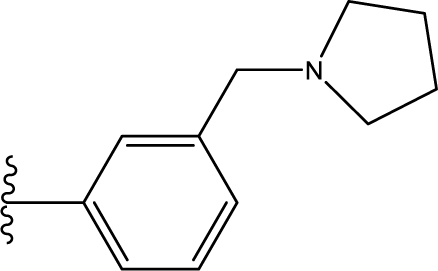		1.959
53	d	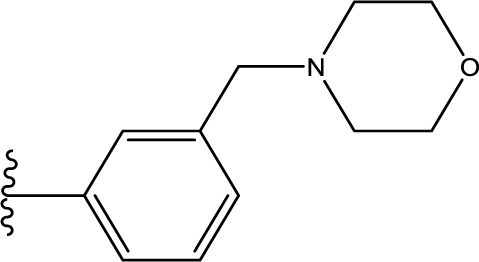		1.244
54	d	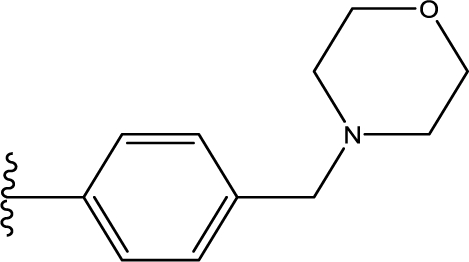		1.180
55	d	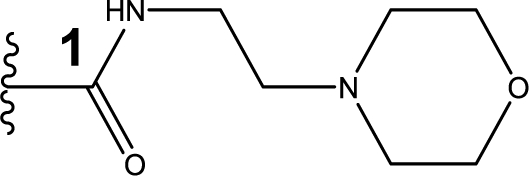		2.301
57	d	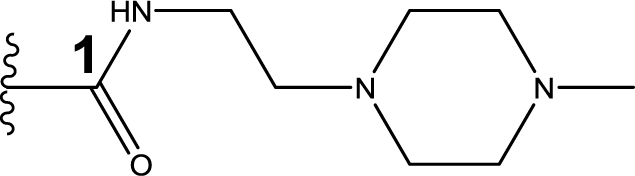		2.301
62	d	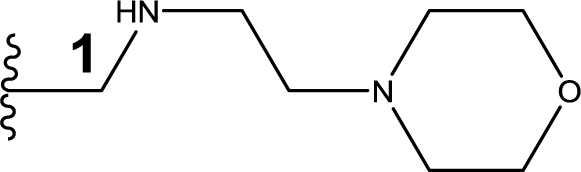		1.658
63	d	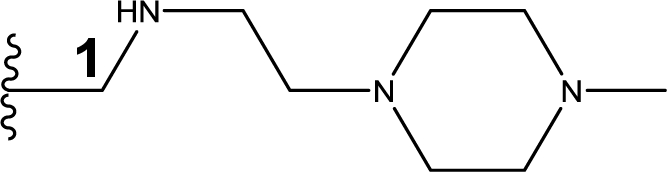		1.081

**Compound**	**Template**	**R_2_**	**R_3_**	**p*IC*_50_ (μM)**

65	e	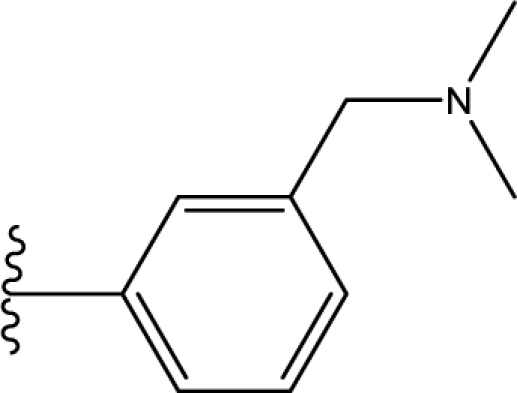	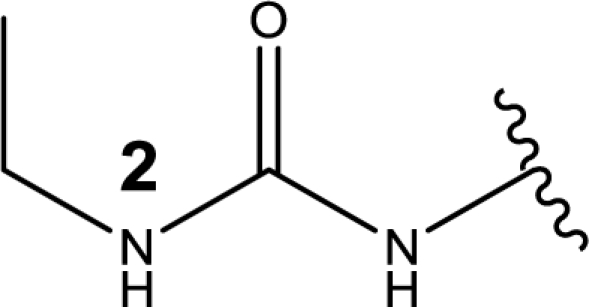	2.523
66	e	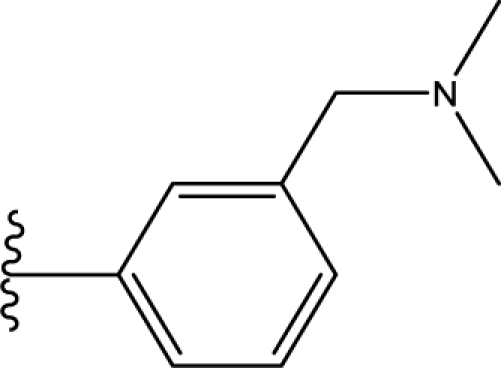	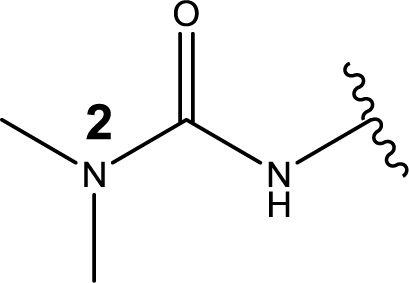	2.301
67	e	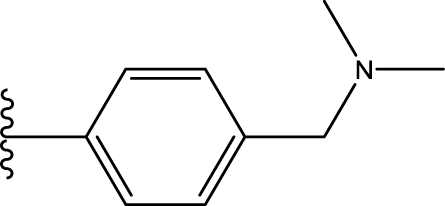	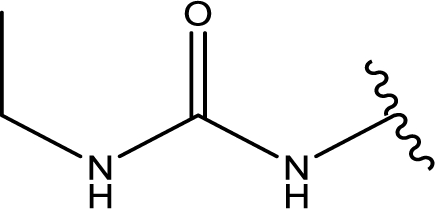	2.770
68	e	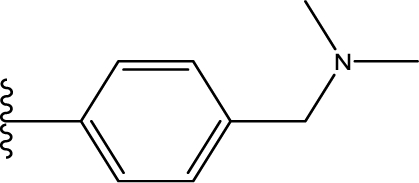	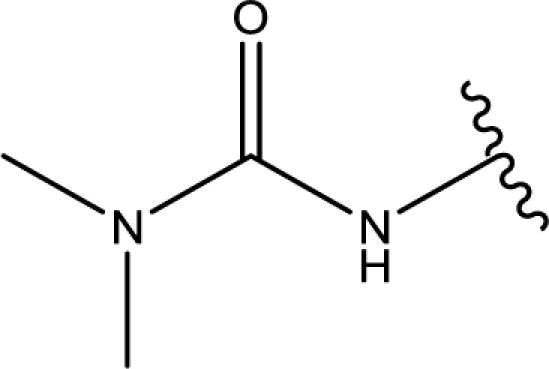	2.444
69	e	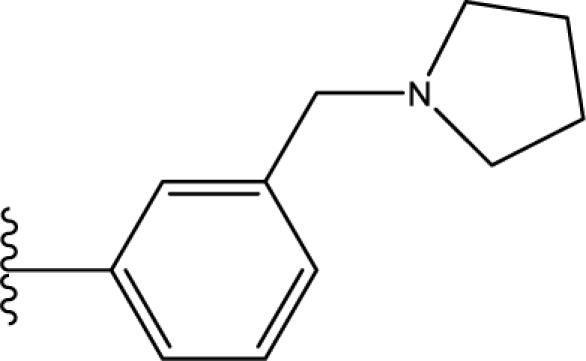	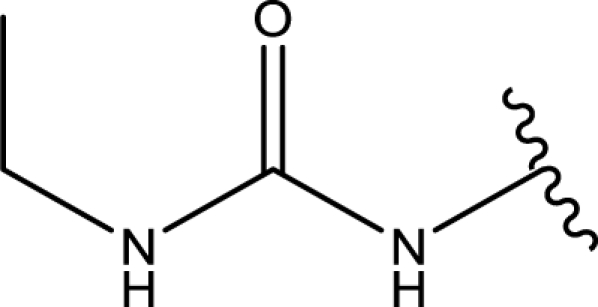	2.854
70	e	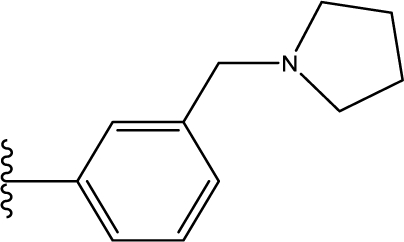	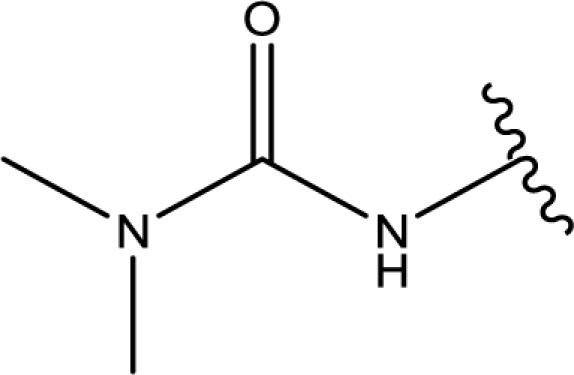	2.678
71^a^	e	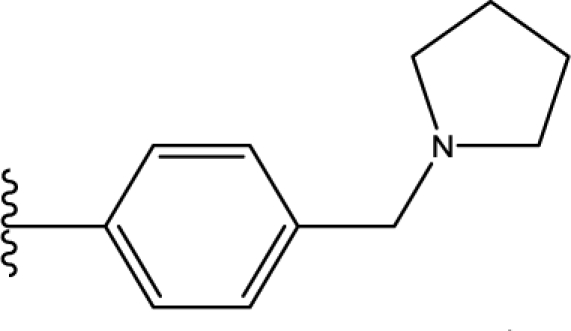	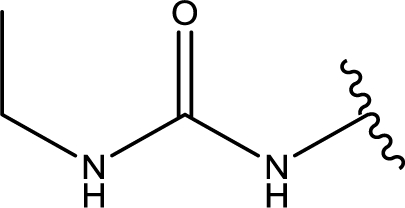	2.824
72	e	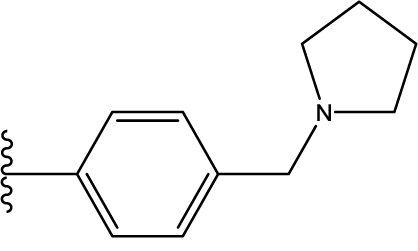	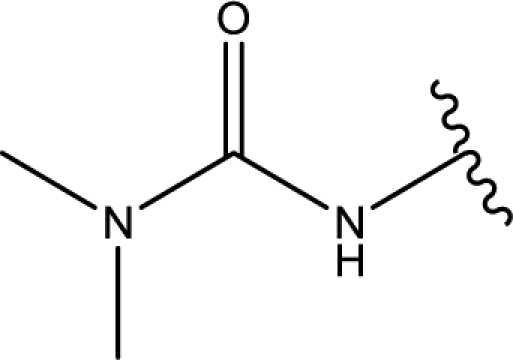	2.347
75	e	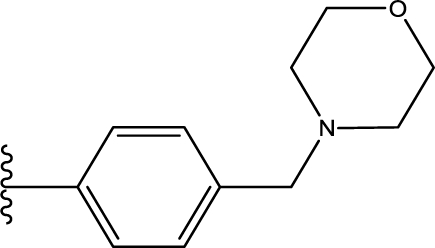	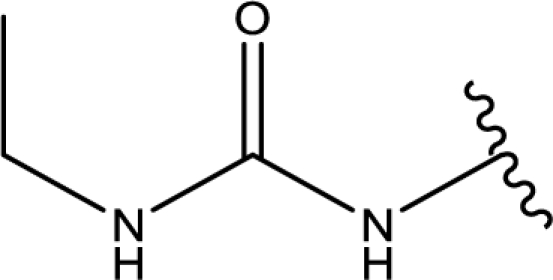	2.328
76	e	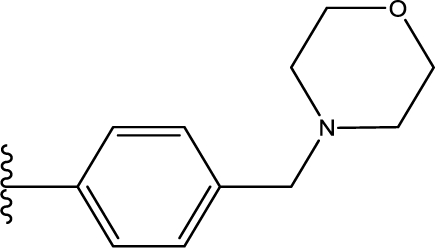	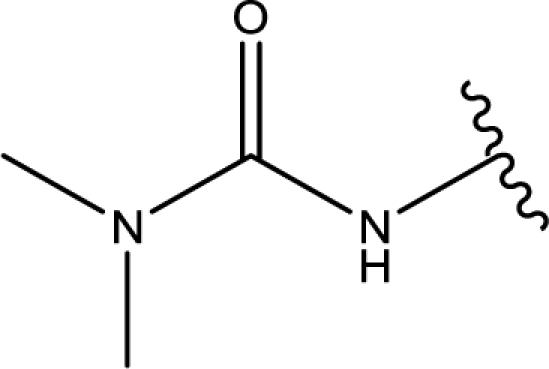	2.092

a Test set molecules,

b Outliers. The common structure for molecular alignment is shown in bold face.

**Table 3.** Representative skeletons and molecular structures of sns-314 derivatives and their binding affinity values (p*IC*_50_). 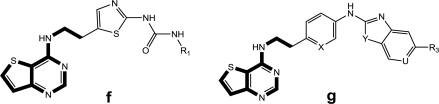

**Compound**	**Template**	**R_1_**	**p*IC*_50_ (μM)**
80	f	−Ph	0.921
82	f	3–F−C_6_H_4_	1.745
88	f	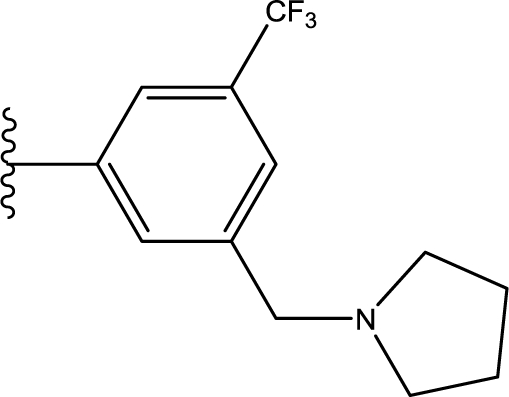	1.658
89^a^	f	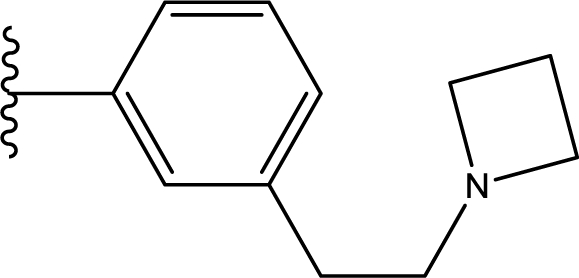	0.921

**Compound**	**Template**	**X**	**Y**	**U**	**R_3_**	**p*IC*_50_ (μM)**
94	g	CH	NH	CH	H	2.000
97	g	CH	NMe	CH	H	0.553
98	g	CH	O	CH	H	0.921
99	g	CH	S	CH	H	−0.398
103^b^	g	N	NH	CH	CF_3_	1.201
105	g	CH	NH	N	CF_3_	2.301

a Test set molecules,

b Outliers. The common structure for molecular alignment is shown in bold face.

**Table 4.** Summary of statistics and field contributions for the top model of each class.

**Parameters**	**MK-0457**	**GSK1070916**	**SNS-314**

	**CoMSIA**	**CoMSIA**	**CoMSIA**

*Q*^2^^a^	0.605	0.52	0.582
*R*^2^_ncv_^b^	0.882	0.904	0.889
*SEE*^c^	0.232	0.215	0.295
*F*^d^	50.159	65.993	28.832
*R*^2^_pred_^e^	0.826	0.798	0.971

*SEP*^f^	0.410	0.482	0.572
PLS components g	3	4	5
*R*^2^_boot_^h^	0.930	0.936	0.921
*SD*_boot_^i^	0.028	0.023	0.032
*SEE*_boot_^j^	0.174	0.172	0.245

**Field contribution**			

Steric	0.323	-	-
Electrostatic	0.677	0.69	-
Hydrophobic	-	-	0.607
Hydrogen-bond-donor	-	0.31	0.393

a Cross-validated correlation coefficient after the leave-one-out procedure;

b Non-cross-validated correlation coefficient;

c Standard error of estimate;

d Ratio of *R*^2^_ncv_ explained to unexplained = *R*^2^_ncv_/(1−*R*^2^_ncv_);

e Predicted correlation coefficient for the test set of compounds;

f Standard error of prediction.

g Optimal number of principal components;

h Average of correlation coefficient for a total of 100 runs of bootstrap analysis;

i Standard deviation of average bootstrap analysis correlation coefficient for 100 runs;

j Average standard error of estimate for a total of 100 runs of bootstrap analysis.
