# Supplementary material for: 3D-QSAR and Molecular Docking Studies on Derivatives of MK-0457, GSK1070916 and SNS-314 as Inhibitors against Aurora B Kinase
Source: Int J Mol Sci. 2010 Nov 2;11(11):4326–47. doi: 10.3390/ijms11114326 (PMC3000085; doi:10.3390/ijms11114326)

Supplementary Data

Table S1. Molecular structures of MK-0457(VX-680) Derivatives and Their Binding Affinity Values (pK_i). The template for molecular alignment is shown in bold face.

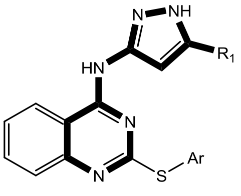

a

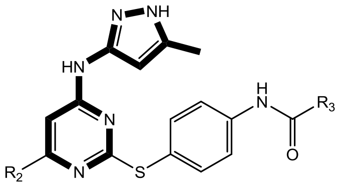

b

Compound	Template	R_1	Ar	Actual pK_i (μM)	Predicted pK_i (μM)	ref
1	a	Me	Ph	0.229	0.258	8
2[a]	a	Me	2-ClPh	0.352	0.208	8
3	a	Me	3-ClPh	0.733	0.384	8
4	a	Me	2,3-DiClPh	0.171	0.340	8
5	a	Me	2-OMe	0.002	0.110	8
6	a	Me	4-OMe	0.240	0.515	8
7	a	Me	3,4-DiOMePh	0.921	0.734	8
8	a	Me	4-($NHSO_2Me$)Ph	0.638	0.962	8
9	a	Me	4-(NHC(O)OtBu)Ph	0.602	0.760	8
10	a	Me	4-(NHC(O)Me)Ph	1.569	1.327	8
11	a	Me	4-(NMeC(O)Me)Ph	0.979	1.051	8
12[a]	a	Me	4-(NHC(O)Et)Ph	1.244	1.331	8
13	a	Me	4-(NHC(O)cPr)Ph	1.292	1.227	8
14	a	cPr	4-(NHC(O)Me)Ph	1.824	1.511	8
		R_2	R_3			8
15	b	H	Me	0.684	0.617	8
16[a]	b	Me	Me	0.658	0.81	8
17[a]	b	Ph	Me	1.081	1.212	8
18	b	Me	Et	0.815	0.855	8
19[a]	b	CyPr	Et	1.229	1.122	8
20[a]	b	tBu	Et	0.939	1.368	8
21	b	Ph	Et	0.839	1.229	8
22	b	3-Py	Et	1.284	0.867	8
23	b	4-Py	Et	1.310	1.143	8
24	b	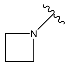	Et	1.638	1.537	8
25	b	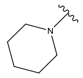	Et	2.097	1.881	8
26	b	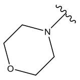	Et	1.854	1.860	8
27	b	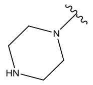	Et	1.745	1.838	8
28	b	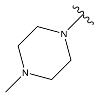	Et	1.699	1.962	8

29[a]	b	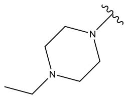	Et	1.602	2.022	8
30[a]	b	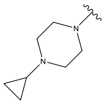	Et	2.022	1.842	8
31	b	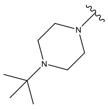	Et	1.959	2.027	8
32	b	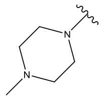	CyPr	1.745	1.855	8

[a]Test set molecules.

Table S2. Molecular structures of GSK1070916 Derivatives and Their Binding Affinity Values (pIC_{50}). The template for molecular alignment is shown in bold face.

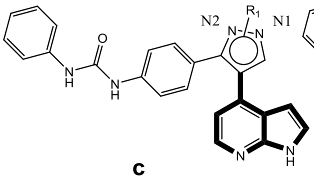

c

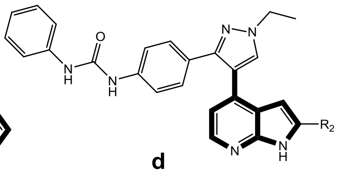

d

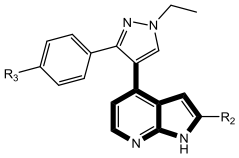

e

Compound	Template	R_1	R_1 position	Actual pK_i(μM)	Predicted pK_i(μM)	ref
33[a]	c	H	N_1	1.495	1.871	9
34	c	Et	N_2	1.602	1.596	9
35[b]	c	Et	N_1	2.699	1.459	9
36	c	tBu	N_2	1.398	1.062	9
37	c	tBu	N_1	1.301	1.392	9
38	c	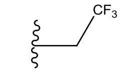	N_1	1.796	1.677	9
39	c	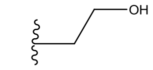	N_1	2.523	2.229	9
40	c	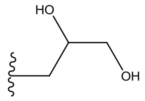	N_1	3.000	3.148	9
41	c	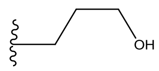	N_1	2.699	2.644	9
42	c	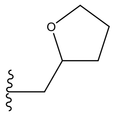	N_1	1.013	1.283	9

43	c	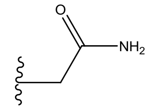	N_1	1.194	1.218	9
44[a]	c	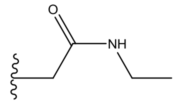	N_1	1.194	0.987	9
45	c	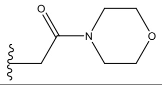	N_1	1.071	0.858	9
		**R_2**				
46	d	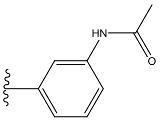		1.357	1.348	9
47[a]	d	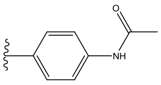		1.357	1.414	9
48[a]	d	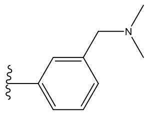		1.699	1.449	9
49	d	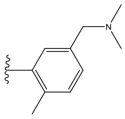		1.222	1.274	9
50	d	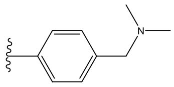		1.638	1.724	9
51[a]	d	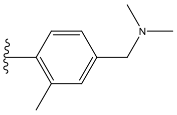		1.585	1.489	9
52[a]	d	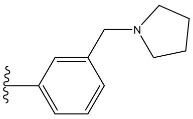		1.959	1.614	9
53	d	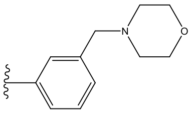		1.244	1.236	9
54	d	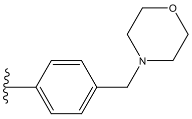		1.180	1.360	9
55	d	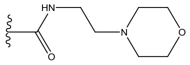		2.301	2.304	9
56	d	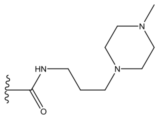		2.097	2.133	9
57	d	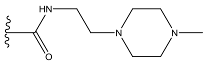		2.301	2.309	9
58[a]	d	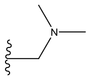		1.237	1.433	9
59	d	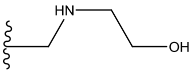		1.337	1.208	9
60	d	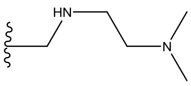		0.559	1.092	9

61	d	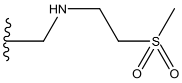		1.620	1.412	9
62	d	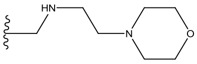		1.658	1.334	9
63	d	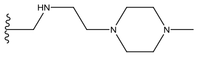		1.081	1.301	9
		**R$_2$**	**R$_3$**			
64	e	H	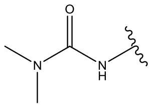	1.585	2.115	9
65	e	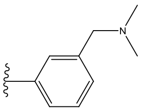	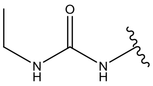	2.523	2.591	9
66	e	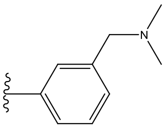	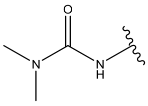	2.301	2.163	9
67	e	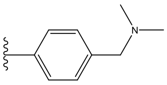	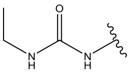	2.770	2.764	9
68	e	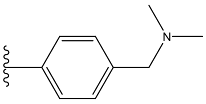	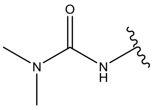	2.444	2.388	9
69	e	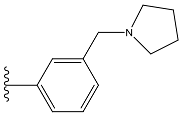	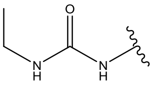	2.854	2.864	9
70	e	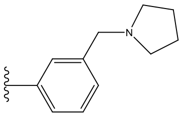	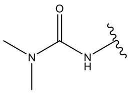	2.678	2.436	9
71[a]	e	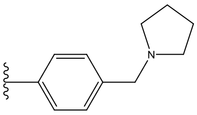	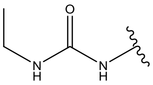	2.824	2.606	9
72	e	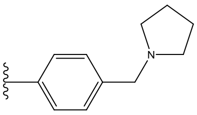	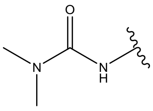	2.347	2.23	9
73[a]	e	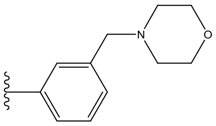	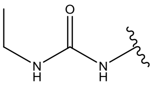	2.301	2.552	9
74[a]	e	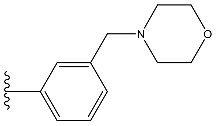	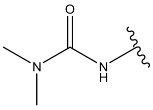	2.328	2.124	9
75	e	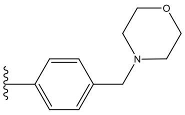	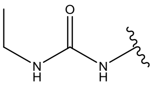	2.328	2.398	9
76	e	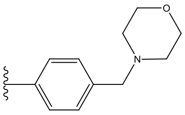	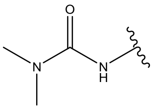	2.092	2.023	9

[a] Test set molecules, [b] Outliers.

Table S3. Molecular structures of SNS-314 Derivatives and Their Binding Affinity Values (pIC_{50}). The template for molecular alignment is shown in bold face.

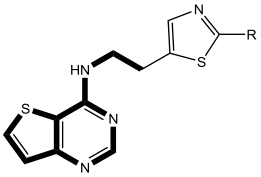

f

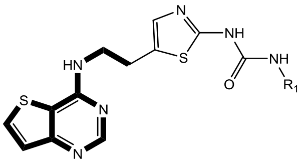

g

Compound	Template	R	Actual pK_i(μM)	Predicted pK_i(μM)	ref
77[a]	f	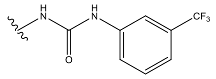	1.854	1.971	10
78	f	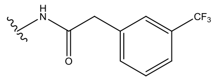	0.237	0.265	10
79	f	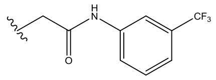	0.355	0.538	10
		R_1			
80	g	-Ph	0.921	1.063	10
81	g	3-Me-C_6H_4	1.268	1.275	10
82	g	3-F-C_6H_4	1.745	1.605	10
83[a]	g	3-Cl-C_6H_4	1.509	1.757	10
84	g	3,4-Cl_2-C6H_3	1.886	1.755	10
85	g	3-Cl-4-F-C_6H_3	1.292	1.273	10
86	g	7-Indolyl	1.721	1.795	10
87[a]	g	Cyclohexyl	1.215	0.952	10
88	g	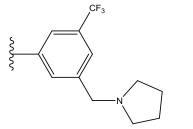	1.658	1.607	10
89[a]	g	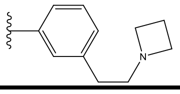	0.921	0.913	10

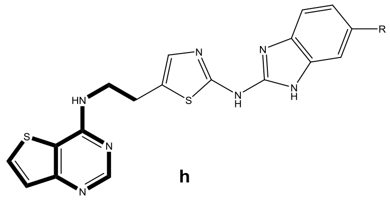

h

Compound	Template	R	Actual pK_i(μM)	Predicted pK_i(μM)	ref
90	h	H	0.377	0.369	4
91	h	CF_3	0.208	0.400	4

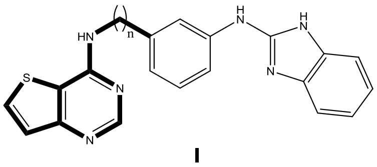

I

Compound	Template	n	Actual pK_i(μM)	Predicted pK_i(μM)	ref
92[a]	I	1	0.237	-0.101	4
93	I	2	-0.146	-0.262	4

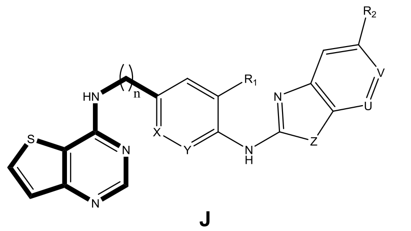

J

Compound	X	Y	Z	U	V	R_1	R_2	Actual pK_i(μM)	Predicted pK_i(μM)	ref
94	CH	CH	NH	CH	CH	H	H	2.000	1.493	4
95	CH	CH	NH	N	CH	H	H	1.081	1.334	4
96	CH	CH	NH	CH	N	H	H	0.854	0.846	4
97	CH	CH	NMe	CH	CH	H	H	0.553	0.342	4
98	CH	CH	O	CH	CH	H	H	0.921	0.461	4
99	CH	CH	S	CH	CH	H	H	-0.398	0.298	4
100	CH	CH	NH	CH	CH	H	CF_3	2.222	2.230	4
101[a]	CH	CH	NH	CH	CH	H	Cl	2.097	2.204	4
102	CH	CH	NH	CH	CH	F	CF_3	1.678	2.189	4
103	N	CH	NH	CH	CH	H	CF_3	1.201	1.046	4
104	CH	N	NH	CH	CH	H	CF_3	0.222	0.127	4
105	CH	CH	NH	N	CH	H	CF_3	2.301	2.067	4
106	CH	CH	NH	N	CH	H	Cl	2.000	2.040	4
107[b]	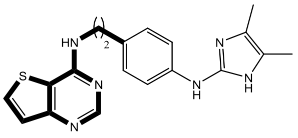							-0.748	1.325	4
108[a]	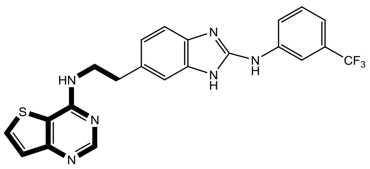							-0.146	-0.273	4

[a] Test set molecules, [b] Outliers.

Table S4. Experimental, Predicted Activities and Residual Values by the Top Model of MK-0457(VX-680) Derivatives.

Compound	pK_i (μM)			
	Actual	Predicted	Residues	ref
1	0.229	0.258	-0.029	8
3	0.733	0.384	0.349	8
4	0.171	0.340	-0.169	8
5	0.002	0.110	-0.108	8
6	0.240	0.515	-0.275	8

7	0.921	0.734	0.187	8
8	0.638	0.962	-0.324	8
9	0.602	0.760	-0.158	8
10	1.569	1.327	0.242	8
11	0.979	1.051	-0.072	8
13	1.292	1.227	0.065	8
14	1.824	1.511	0.313	8
15	0.684	0.617	0.067	8
18	0.815	0.855	-0.040	8
21	0.839	1.229	-0.390	8
22	1.284	0.867	0.417	8
23	1.310	1.143	0.167	8
24	1.638	1.537	0.101	8
25	2.097	1.881	0.216	8
26	1.854	1.860	-0.006	8
27	1.745	1.838	-0.093	8
28	1.699	1.962	-0.263	8
31	1.959	2.027	-0.068	8
32	1.745	1.855	-0.110	8
Average			0.001	
		**Test set**		
2	0.352	0.208	0.144	8
12	1.244	1.331	-0.087	8
16	0.658	0.81	-0.152	8
17	1.081	1.212	-0.131	8
19	1.229	1.122	0.107	8
20	0.939	1.368	-0.429	8
29	1.602	2.022	-0.420	8
30	2.022	1.842	0.180	8
Average			-0.098	

Table S5. Experimental, Predicted Activities and Residual Values by the Top Model of GSK1070916 Derivatives Derivatives.

**Compound**	**pIC_{50} (μM)**			
	**Actual**	**Predicted**	**Residues**	**ref**
34	1.602	1.596	0.006	9
36	1.398	1.062	0.336	9
37	1.301	1.392	-0.091	9
38	1.796	1.677	0.119	9
39	2.523	2.229	0.294	9
40	3.000	3.148	-0.148	9
41	2.699	2.644	0.055	9
42	1.013	1.283	-0.270	9
43	1.194	1.218	-0.024	9
45	1.071	0.858	0.213	9
46	1.357	1.348	0.009	9

49	1.222	1.274	-0.052	9
50	1.638	1.724	-0.086	9
53	1.244	1.236	0.008	9
54	1.180	1.360	-0.180	9
55	2.301	2.304	-0.003	9
56	2.097	2.133	-0.036	9
57	2.301	2.309	-0.008	9
59	1.337	1.208	0.129	9
60	0.559	1.092	-0.533	9
61	1.620	1.412	0.208	9
62	1.658	1.334	0.324	9
63	1.081	1.301	-0.220	9
64	1.585	2.115	-0.530	9
65	2.523	2.591	-0.068	9
66	2.301	2.163	0.138	9
67	2.770	2.764	0.006	9
68	2.444	2.388	0.056	9
69	2.854	2.864	-0.010	9
70	2.678	2.436	0.242	9
72	2.347	2.23	0.117	9
75	2.328	2.398	-0.070	9
76	2.092	2.023	0.069	9
35 [a]	2.699	1.459	1.240	9
Average			0.000	
**Test set**				
33	1.495	1.871	-0.376	9
44	1.194	0.987	0.207	9
47	1.357	1.414	-0.057	9
48	1.699	1.449	0.250	9
51	1.585	1.489	0.096	9
52	1.959	1.614	0.345	9
58	1.237	1.433	-0.196	9
71	2.824	2.606	0.218	9
73	2.301	2.552	-0.251	9
74	2.328	2.124	0.204	9
Average			0.044	

[a] Outliers.

Table S6. Experimental, Predicted Activities and Residual Values by the Top Model of SNS-314 Derivatives Derivatives.

**Compound**	**pIC_{50} (μM)**			
	**Actual**	**Predicted**	**Residues**	**ref**
**Training set**				
78	0.237	0.265	-0.028	10
79	0.355	0.538	-0.183	10
80	0.921	1.063	-0.142	10

81	1.268	1.275	-0.007	10
82	1.745	1.605	0.140	10
84	1.886	1.755	0.131	10
85	1.292	1.273	0.019	10
86	1.721	1.795	-0.074	10
88	1.658	1.607	0.051	10
90	0.377	0.369	0.008	4
91	0.208	0.400	-0.192	4
93	-0.146	-0.262	0.116	4
94	2.000	1.493	0.507	4
95	1.081	1.334	-0.253	4
96	0.854	0.846	0.008	4
97	0.553	0.342	0.211	4
98	0.921	0.461	0.460	4
99	-0.398	0.298	-0.696	4
100	2.222	2.230	-0.008	4
102	1.678	2.189	-0.511	4
103	1.201	1.046	0.155	4
104	0.222	0.127	0.095	4
105	2.301	2.067	0.234	4
106	2.000	2.040	-0.040	4
107 [a]	-0.748	1.325	-2.073	4
Average			0.015	
		**Test set**		
77	1.854	1.971	-0.117	10
83	1.509	1.757	-0.248	10
87	1.215	0.952	0.263	10
89	0.921	0.913	0.008	10
92	0.237	-0.101	0.338	4
101	2.097	2.204	-0.107	4
108	-0.146	-0.273	0.127	4
Average			0.038	

[a] Outliers.

Table S7. Combinations of different CoMSIA/CoMFA fields and their results[a] for derivates of MK-0457.

**Field(s)**	r^2_{cv}	**comp.**	***SEP***	r^2_{ncv}	***SEE***	***F* value**	**Contribution**				
							S	E	H	D	A
S	0.570	1	0.425	0.656	0.379	41.883	1.00	-	-	-	-
E	0.590	3	0.433	0.883	0.232	50.260	-	1.00	-	-	-
H	0.565	1	0.426	0.667	0.372	44.145	-	-	1.00	-	-
D	0.314	1	0.534	0.470	0.470	19.506	-	-	-	1.00	-
A	0.346	1	0.522	0.515	0.449	23.364	-	-	-	-	1.00
**SE**	**0.605**	**3**	**0.410**	**0.883**	**0.232**	**50.159**	**0.33**	**0.67**	-	-	-
SH	0.585	1	0.416	0.682	0.364	47.205	0.29	-	0.71	-	-
SD	0.460	1	0.474	0.597	0.410	32.549	0.38	-	-	0.62	

SA	0.533	3	0.462	0.824	0.284	31.274	0.53	-	-	-	0.47
EH	0.577	1	0.420	0.688	0.361	48.404	-	0.43	0.57	-	-
ED	0.572	7	0.495	0.974	0.122	85.018	-	0.75	0.25	-	-
EA	0.535	7	0.516	0.982	0.103	121.763	-	0.62	0.38	-	-
HD	0.523	1	0.445	0.644	0.385	39.745	-	-	0.61	0.39	-
HA	0.528	1	0.443	0.651	0.381	40.999	-	-	0.60	0.40	-
DA	0.335	1	0.526	0.500	0.456	21.997	-	-	-	0.49	0.51
SEH	0.591	1	0.412	0.696	0.355	50.483	0.19	0.34	0.47	-	-
SED	0.596	4	0.441	0.934	0.178	67.324	0.24	0.55	-	0.21	-
SEA	0.599	7	0.479	0.979	0.109	108.468	0.22	0.45	-	-	0.33
SHD	0.550	1	0.433	0.663	0.374	43.372	0.20	-	0.49	0.31	-
SHA	0.554	1	0.431	0.669	0.371	44.515	0.20	-	0.48	-	0.32
SDA	0.501	3	0.478	0.832	0.277	33.118	0.37	-	-	0.25	0.38
EHD	0.545	1	0.435	0.666	0.373	43.894	-	0.31	-	0.42	0.27
EHA	0.546	1	0.435	0.669	0.371	44.427	0.31	-	0.41	-	0.28
EDA	0.521	3	0.468	0.874	0.240	46.193	-	0.53	-	0.22	0.24
HDA	0.485	1	0.463	0.619	0.398	35.725	-	-	0.43	0.28	0.29
SEHD	0.563	1	0.427	0.678	0.366	46.421	0.14	0.27	0.36	0.23	-
SEHA	0.564	1	0.426	0.681	0.364	46.982	0.14	0.26	0.36	-	0.24
SHDA	0.515	1	0.449	0.642	0.386	39.413	0.15	-	0.37	0.24	0.24
SEDA	0.587	3	0.435	0.900	0.214	59.687	0.21	0.40	-	0.17	0.22
EHDA	0.513	1	0.450	0.644	0.385	39.774	-	0.24	0.33	0.21	0.22
SEHDA	0.533	1	0.441	0.659	0.377	42.515	0.12	0.21	0.29	0.19	0.19
CoMFA	0.604	8	0.492	0.992	0.071	226.450	0.62	0.38	-	-	-

[a]Abbreviations: S (steric); E (electrostatic); H (hydrophobic); D (H-bond donor); A (H-bond acceptor).

Table S8. Combinations of different CoMSIA/CoMFA fields and their results[a] for derivates of GSK1070916

Field(s)	r^2_{cv}	comp.	*SEP*	r^2_{ncv}	*SEE*	*F* value	Contribution				
							S	E	H	D	A
S	0.114	5	0.666	0.725	0.371	14.232	1.00	-	-	-	-
E	0.213	1	0.586	0.423	0.502	22.717	-	1.00	-	-	-
H	0.196	4	0.623	0.790	0.319	26.350	-	-	1.00	-	-
D	0.29	7	0.620	0.650	0.435	6.634	-	-	-	1.00	-
A	-0.198	3	0.748	0.545	0.461	11.576	-	-	-	-	1.00
SE	0.178	3	0.619	0.742	0.347	27.788	0.27	0.73	-	-	-
SH	0.196	4	0.623	0.751	0.347	21.136	0..26	-	0.74	-	-
SD	0.483	5	0.509	0.858	0.267	32.584	0.48	-	-	0.52	-
SA	0.371	9	0.608	0.958	0.156	58.948	0.39	-	-	-	0.61
EH	0.25	4	0.602	0.851	0.269	39.897	-	0.52	0.48	-	-
**ED**	**0.520**	**4**	**0.482**	**0.904**	**0.215**	**65.993**	-	**0.69**	-	**0.31**	-
EA	0.361	9	0.613	0.983	0.100	148.595	-	0.49	-	-	0.51
HD	0.438	4	0.521	0.848	0.271	38.972	-	-	0.65	0.35	-
HA	0.421	9	0.584	0.978	0.113	114.506	-	-	0.45	-	0.55
DA	0.205	9	0.684	0.854	0.293	14.957	-	-	-	0.27	0.73

SEH	0.245	4	0.604	0.833	0.284	34.873	0.15	0.44	0.41	-	-
SED	0.451	5	0.525	0.911	0.211	55.258	0.19	0.52	-	0.29	-
SEA	0.404	9	0.592	0.984	0.098	154.478	0.18	0.40	-	-	0.42
SHD	0.393	4	0.542	0.833	0.285	34.793	0.19	-	0.51	0.30	-
SHA	0.424	9	0.582	0.979	0.112	116.462	0.12	-	0.38	-	0.50
SDA	0.517	9	0.533	0.965	0.144	69.991	0.26	-	-	0.22	0.52
EHD	0.398	4	0.540	0.875	0.246	48.786	-	0.41	0.38	0.21	-
EHA	0.365	9	0.611	0.983	0.100	147.650	-	0.27	0.32	-	0.41
EDA	0.488	9	0.549	0.984	0.097	158.492	-	0.40	-	0.18	0.42
HDA	0.506	9	0.539	0.978	0.115	111.983	-	-	0.38	0.16	0.46
SEHD	0.377	4	0.549	0.864	0.256	44.477	0.12	0.35	0.33	0.20	-
SEHA	0.382	9	0.603	0.983	0.100	146.595	0.09	0.25	0.28	-	0.38
SHDA	0.475	9	0.556	0.977	0.115	111.015	0.10	-	0.32	0.15	0.43
SEDA	0.477	9	0.555	0.986	0.090	181.403	0.13	0.33	-	0.16	0.38
EHDA	0.439	7	0.551	0.966	0.137	100.089	-	0.25	0.27	0.13	0.35
SEHDA	0.420	6	0.550	0.958	0.149	97.763	0.08	0.22	0.24	0.13	0.33
CoMFA	0.249	9	0.665	0.989	0.079	239.811	0.57	0.43	-	-	-

[a]Abbreviations: S (steric); E (electrostatic); H (hydrophobic); D (H-bond donor); A (H-bond acceptor).

Table S9. Combinations of different CoMSIA/CoMFA fields and their results[a] for derivates of SNS-314.

**Field(s)**	r^2_{cv}	**comp.**	***SEP***	r^2_{ncv}	***SEE***	***F* value**	**Contribution**				
							**S**	**E**	**H**	**D**	**A**
S	0.081	1	0.767	0.257	0.689	7.610	1.00	-	-	-	-
E	-0.018	3	0.846	0.620	0.517	10.868	-	1.00	-	-	-
H	0.462	4	0.631	0.804	0.381	19.524	-	-	1.00	-	-
D	0.379	6	0.717	0.672	0.521	5.800	-	-	-	1.00	-
A	-0.099	2	0.858	0.335	0.667	5.301	-	-	-	-	1.00
SE	0.069	2	0.790	0.555	0.546	13.087	0.22	0.77	-	-	-
SH	0.466	4	0.629	0.806	0.379	19.702	0.18	-	0.82	-	-
SD	0.297	3	0.703	0.590	0.537	9.611	0.15	-	-	0.85	-
SA	0.132	3	0.781	0.532	0.574	7.580	0.35	-	-	-	0.65
EH	0.312	4	0.714	0.840	0.344	24.935	-	0.46	0.54	-	-
ED	0.346	2	0.662	0.653	0.482	19.728	-	0.50	-	0.50	-
EA	0.003	3	0.838	0.600	0.531	9.994	-	0.62	-	-	0.38
**HD**	**0.582**	**5**	**0.572**	**0.889**	**0.295**	**28.832**	-	-	**0.61**	**0.39**	-
HA	0.300	3	0.702	0.729	0.437	17.903	-	-	0.71	-	0.29
DA	0.319	3	0.692	0.681	0.473	14.251	-	-	-	0.59	0.41
SEH	0.322	4	0.709	0.817	0.368	21.263	0.09	0.42	0.49	-	-
SED	0.386	2	0.641	0.635	0.495	18.255	0.10	0.46	-	0.44	-
SEA	0.104	3	0.794	0.633	0.508	11.501	0.15	0.52	-	-	0.33
SHD	0.553	2	0.547	0.755	0.405	32.433	0.08	-	0.50	0.42	-
SHA	0.333	4	0.703	0.783	0.401	17.109	0.15	-	0.66	-	0.19
SDA	0.387	2	0.641	0.686	0.459	22.924	0.11	-	-	0.51	0.38
EHD	0.418	2	0.625	0.703	0.446	24.864	-	0.30	0.34	0.36	-

EHA	0.239	4	0.751	0.808	0.378	19.930	-	0.38	0.48	-	0.14
EDA	0.283	3	0.710	0.745	0.424	19.429	-	0.35	-	0.41	0.24
HDA	0.437	3	0.629	0.765	0.407	21.673	-	-	0.40	0.37	0.23
SEHD	0.430	2	0.618	0.716	0.436	26.519	0.07	0.28	0.33	0.32	-
SEHA	0.267	4	0.737	0.812	0.373	20.500	0.09	0.35	0.44	-	0.12
SHDA	0.440	3	0.628	0.762	0.409	21.341	0.07	-	0.37	0.34	0.22
SEDA	0.311	2	0.679	0.662	0.476	20.580	0.09	0.32	-	0.38	0.21
EHDA	0.440	3	0.628	0.762	0.409	21.341	0.07	-	0.37	0.34	0.22
SEHDA	0.369	4	0.684	0.856	0.326	28.321	0.05	0.23	0.30	0.30	0.12
CoMFA	0.079	3	0.805	0.707	0.454	16.053	0.45	0.55	-	-	-

[a]Abbreviations: S (steric); E (electrostatic); H (hydrophobic); D (H-bond donor); A (H-bond acceptor).

Figure S1. This picture shows the schematics for the superposition of 2BFX template (green ribbon) and the Aurora B model structure (red ribbon) from homology modeling.

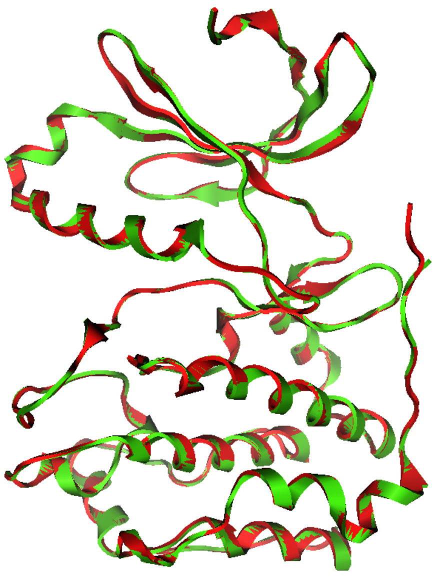

Figure S2. 3-D view of all aligned derivatives of MK-0457(VX-680), GSK10709106 and SNS-314, respectively, as prepared by the alignment algorithm.

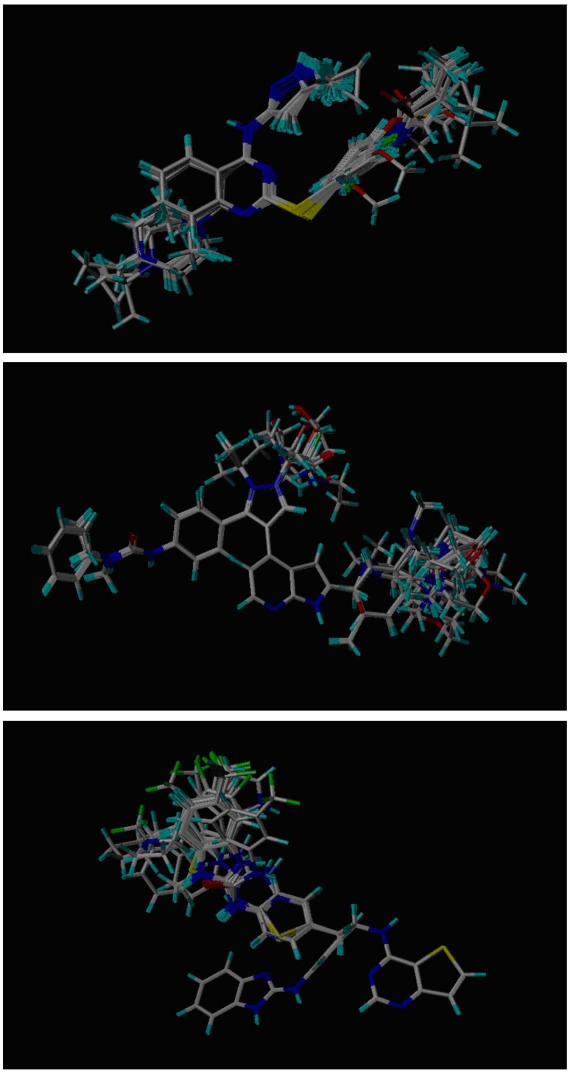

Supplement: Supplementary file 1 [file ijms-11-04326-s001.pdf]
